# Supplementary material for: Qualitative study exploring health care professionals’ perceptions of providing rehabilitation for people with advanced dementia
Source: BMJ Open. 2023 Jul 31;13(7):e072432. doi: 10.1136/bmjopen-2023-072432 (PMC10391829; doi:10.1136/bmjopen-2023-072432)
Supplement: Supplementary data [file bmjopen-2023-072432supp002.pdf]

Physical health rehabilitation interventions for people with advanced dementia

TOPIC GUIDE

Researcher to introduce self and explain process of focus group, group rules, confidentiality, permission to record, right to withdraw.

Topic	Prompts
**Introduction – each member**<br><br>Please can you tell me a bit about your experience of working with people with advanced dementia and any challenges that you experience supporting them	Profession<br><br>Interest in PwD<br><br>System/organisational pressures<br><br>Individual patient difficulties<br><br>Carer
**Treatments**<br><br>What things are important to ensure that a treatment with a person with advanced dementia is effective?	Who present?<br><br>Involvement of family/carers<br><br>Duration<br><br>Type of intervention<br><br>Patient outcomes / QoL<br><br>Daily routines
**Location**<br><br>What are the key considerations when providing healthcare interventions in a nursing home compared to at home?	Environment<br><br>Formal carers – engagement, education, knowledge<br><br>Equipment
**Communication**<br><br>What factors are important to consider when communicating with a person with advanced dementia?	Body language<br><br>Tone

	Language
**Training/education**<br><br>Therapists with a variety of experience will be treating people with advanced dementia, therefore what education or skills are needed to be able to treat people with advanced dementia?	Undergraduate learning<br><br>Formal learning<br><br>Experiential learning

Is there something else that I have not asked you about, that you would like to tell me about?

Thank you
