# Supplementary material for: Qualitative study exploring health care professionals’ perceptions of providing rehabilitation for people with advanced dementia
Source: BMJ Open. 2023 Jul 31;13(7):e072432. doi: 10.1136/bmjopen-2023-072432 (PMC10391829; doi:10.1136/bmjopen-2023-072432)
Supplement: Author's manuscript [file bmjopen-2023-072432.draft_revisions.pdf]

BMJ Open is committed to open peer review. As part of this commitment we make the peer review history of every article we publish publicly available.

When an article is published we post the peer reviewers' comments and the authors' responses online. We also post the versions of the paper that were used during peer review. These are the versions that the peer review comments apply to.

The versions of the paper that follow are the versions that were submitted during the peer review process. They are not the versions of record or the final published versions. They should not be cited or distributed as the published version of this manuscript.

BMJ Open is an open access journal and the full, final, typeset and author-corrected version of record of the manuscript is available on our site with no access controls, subscription charges or pay-per-view fees (http://bmjopen.bmj.com).

If you have any questions on BMJ Open's open peer review process please email info.bmjopen@bmj.com

BMJ Open

What does it mean to provide rehabilitation for a person with advanced dementia?

Journal:	*BMJ Open*
Manuscript ID	bmjopen-2023-072432
Article Type:	Original research
Date Submitted by the Author:	07-Feb-2023
Complete List of Authors:	Hall, Abigail J.; University of Exeter<br>Manning, Fay; University of Exeter Medical School, Exeter Medical School<br>Goodwin, Victoria; University of Exeter, PenCLAHRC
Keywords:	Dementia, REHABILITATION MEDICINE, QUALITATIVE RESEARCH

SCHOLARONE™
Manuscripts

I, the Submitting Author has the right to grant and does grant on behalf of all authors of the Work (as defined in the below author licence), an exclusive licence and/or a non-exclusive licence for contributions from authors who are: i) UK Crown employees; ii) where BMJ has agreed a CC-BY licence shall apply, and/or iii) in accordance with the terms applicable for US Federal Government officers or employees acting as part of their official duties; on a worldwide, perpetual, irrevocable, royalty-free basis to BMJ Publishing Group Ltd ("BMJ") its licensees and where the relevant Journal is co-owned by BMJ to the co-owners of the Journal, to publish the Work in this journal and any other BMJ products and to exploit all rights, as set out in our licence.

The Submitting Author accepts and understands that any supply made under these terms is made by BMJ to the Submitting Author unless you are acting as an employee on behalf of your employer or a postgraduate student of an affiliated institution which is paying any applicable article publishing charge ("APC") for Open Access articles. Where the Submitting Author wishes to make the Work available on an Open Access basis (and intends to pay the relevant APC), the terms of reuse of such Open Access shall be governed by a Creative Commons licence – details of these licences and which Creative Commons licence will apply to this Work are set out in our licence referred to above.

Other than as permitted in any relevant BMJ Author's Self Archiving Policies, I confirm this Work has not been accepted for publication elsewhere, is not being considered for publication elsewhere and does not duplicate material already published. I confirm all authors consent to publication of this Work and authorise the granting of this licence.

For peer review only

What does it mean to provide rehabilitation for a person with advanced dementia?

Abigail J Hall (corresponding author) BSc, MSc, PhD, MCSP

University of Exeter, Heavitree Road, Exeter, EX1 2LU

a.hall4@exeter.ac.uk

Fay Manning BSc, MSc, PhD

University of Exeter, Heavitree Road, Exeter, EX1 2LU

Victoria A Goodwin, MBE, BSc, MSc, PhD, FCSP,

University of Exeter, Heavitree Road, Exeter, EX1 2LU

For peer review only

Abstract

Objectives

The aims of the study were to explore the theoretical basis of health care professionals providing and delivering rehabilitation interventions for people with advanced dementia.

Design

Qualitative study with three focus groups

Participants

20 healthcare professionals who were experts in treating and rehabilitating people with advanced dementia were recruited. Participants needed to have significant experience of treating people with dementia daily.

Results

Three overarching themes were developed following analysis. The first explores how the culture of an organisation affects a person with advanced dementia as well as the healthcare professional. Knowledge was a key consideration and was closely linked to the personal values of the healthcare professional, which formed the final theme. The interrelationship of these three factors influenced the outcomes for the person with dementia.

Conclusions

Effective interventions for people with advanced dementia requires the healthcare professional to have the knowledge and personal values to support the person, but the culture of the organisation is also key to ensure that the healthcare professional is able to demonstrate these personal values and knowledge effectively.

Strengths and limitations

- We recognise that this was a small-scale qualitative study with a limited range of healthcare professionals taking part, however, sampling with deliberately specific and engaged HCPs who were specialists in treating people with very advanced dementia – of which there is a limited sample of people due to the specialist nature of interventions.
- The methods of data collection and analysis were robust and followed clear guidance for undertaking such qualitative work.

For peer review only

- The study was reported using COREQ to ensure clear and transparent methods and analysis.

Background

Dementia is a neurological disorder resulting in physical and cognitive decline, found typically in older adults [1, 2]. It is estimated that by 2040, there will be 1.2 million people with dementia living in the UK – a 57% increase from 2016 [3], suggesting that dementia will become a national – and global – challenge to healthcare services. Advanced dementia is characterised by profound communication, memory and physical disturbances [4], which often result in significant impairments in a person's ability to undertake activities of daily living and a perceived decline in quality of life [5].

A variety of healthcare professionals (HCPs) can be involved in trying to reduce the physical declines associated with advanced dementia, including physiotherapists and occupational therapists. However, there remains little evidence to support physical interventions for people with dementia [6-10]. This lack of evidence is particularly evident for people with more severe dementia, where physical interventions to improve physical ability – or reduce the speed of decline – are largely not understood [6].

Authors have suggested different models and theories to understand the complexities of rehabilitation [11], but all require an understanding of the concepts of health, illness and the influence of the 'body'. Defining illness could depend on the concept of what it means to be 'healthy', as illness could be considered a deviation from this 'healthy' norm [12]. However the concept of what constitutes health may vary between cultures [13] and also between professions and individuals. Thus, the aims of the study were to explore the theoretical basis of HCPs providing and delivering rehabilitation interventions for people with advanced dementia.

Methods/design

During August and September 2022, three semi-structured focus groups were undertaken with a range of HCPs. These health professionals were all experts in the management or rehabilitation of people living with dementia. The focus groups were undertaken virtually to reduce barriers due to geographical limitations and enabled international experts to contribute. The study has been reported according to the COREQ guidelines (appendix 1).

Setting and participants

A purposive sampling strategy was employed to seek HCPs who specialised in working with people with advanced dementia. A targeted social media advert sought professionals with advanced skills working with people with advanced dementia and pre-existing networks were drawn upon to recruit participants. Some of these participants were known to the researchers in a professional capacity due to previous work undertaken in this area. This did not affect the data collection.

For peer review only

Inclusion criteria;

- Specialist working with people with dementia as a physiotherapist, occupational therapist, psychologist, nurse, or other related profession

A patient, public, involvement and engagement (PPIE) group was formed as part of the wider project and their aim was to ensure that the patient and public voice was represented in the design and conduct of the research in order to ensure that it was clinically relevant. Representation from this group was sought for each focus group, however due to practical challenges they were unable to join the focus groups. Therefore, discussion with PPIE representation followed the data collection phase and contributed to the analysis of results. Nobody other than the participants, and the researchers were present during the data collection.

Participants who responded to the social media advert or initial email were sent a follow up email with the participant information sheet attached. At this point they were offered an opportunity to discuss the study further if they had any particular questions. If they were happy to take part in the study, they were asked to provide informed consent prior to the commencement of the focus group. Participants were informed that they were under no obligation to participate in the focus group and they may withdraw from it at any time, without any negative consequence, up until the point where the data were fully anonymised.

Provided the participant met the inclusion criteria, all participants who consented to take part were invited to join a focus group. Recruitment continued until sufficient data was obtained to be able to answer the aim of the study and no new data was being generated.

Data collection

The focus groups aimed to explore the challenges and techniques required to effectively manage people with more advanced dementia, while also considering the theoretical basis of rehabilitation for this population. The focus groups were facilitated by two experienced qualitative researchers (AH and FM). FM acted as a 'second facilitator/observer', taking notes and observations pertaining to interaction of the group. Both researchers have undertaken training courses pertaining to qualitative analysis and have published multiple qualitative papers.

Microsoft Teams was used to undertake the focus groups and they were recorded and automatically transcribed. The transcription function has been used by the researcher and found to be accurate, although the transcripts were all checked for accuracy. The focus groups lasted for a maximum of 90 minutes and included time for participants to introduce themselves and their roles to other members of the group. The participants were informed that this study formed part of a larger piece of work aiming to develop interventions for people with advanced dementia. A semi-structured guide (supplementary data) was used to guide the conversation of the focus groups. The topic guide was not piloted prior to the initial focus group as it was anticipated that it could be adapted for subsequent focus groups if needed. This was not required and the same topic guide was used for all interviews. The data was anonymised so that individual participants could not be identified, and all participants consented to their data being transcribed and analysed. Transcripts were not reviewed

For peer review only

by participants. The data collection from the focus groups provided rich and detailed data and no further follow-up interviews were deemed necessary.

Patient and Public Involvement (PPI)

A patient and public involvement (PPI) group was developed to help inform the research undertaken during this study and as part of a wider programme of work. The group has been evolving and developing and now consists of people with a variety of experiences of dementia.

Due to Covid-19 restrictions, it became evident that a face to face meeting with the group was not feasible. Therefore, the group was consulted individually and then virtually as a group to determine how they would prefer to communicate regarding the project.

Initial discussions focused on the challenges they had experienced with getting rehabilitation for people who have dementia – especially when the person had advanced dementia or was living in a care home. Several themes generated from this discussion formed part of the focus group discussion. Due to practical challenges, none of the PPI group were able to participate in the focus groups, but discussion was had with representatives following the groups and helped guide the analysis and results.

Analysis

Analysis commenced after the first focus group was completed and continued through the following focus groups. As the data collection and initial analysis ran simultaneously arising themes or gaps could be probed accordingly in the remaining focus groups.

NVivo 11 (QSR International) was used to organize and code the data and facilitated a process of reflexive thematic analysis to be undertaken as suggested by Braun and Clarke [14]. Familiarisation of the data was undertaken be reading and re-reading the transcripts. A process of systematic open coding was then commenced and initial ideas for themes were noted. Following this open coding, initial themes were generated, discussed and explored with the wider research team. These discussions lead to further refinement of the themes, and definitions of the themes to be written to ensure clarity between the research team. This process was an iterative process and resulted in several refinements of named themes. These themes were then discussed with a member of the patient and public involvement and engagement (PPIE) group who were recruited to support and collaborate on the overarching project.

Ethics

Ethical approval was obtained from the University of Exeter's College of Medicine and Health Research Ethics Committee (reference 515745).

Findings

For peer review only

20 healthcare professionals (HCPs) took part in three focus groups (Table 1). Only one person who had initially expressed an interest in taking part did not reply to the meeting invite and therefore did not take part. There were no withdrawals from the study at any stages. The HCPs reported an average of eighteen years of experience and an average of thirteen years specializing specifically with people with dementia. The settings of their experience were broad, including in-patient settings, out-patient clinics, patients' own homes and residential/nursing settings.

Table 1 - characteristics of participants (n=20)

**Sex**	**Female**	**19**
	Male	1
**Profession**	Physiotherapist	14
	Occupational Therapist	3
	Nurse	2
	Other	1
**NHS Grade / Banding**	6	6
	7	8
	8+	2
	N/A	4
**Years of clinical experience**	0-5	1
	6 - 10	2
	11-15	1
	16 - 20	3
	21 - 25	6
	26 - 30	2
	30+	3
	N/A	2
**Years of experience working with people with advanced dementia**		
	0-5	4
	6 - 10	2
	11-15	6
	16 - 20	5

For peer review only

	21 - 25	2
	26 - 30	1
	30+	0
**Location**	England	13
	Wales	2
	Scotland	4
	Denmark	1
**% of caseload of people with dementia**		
	0 - 25	1
	26 - 50	1
	51 - 75	6
	76 - 100	8
	N/A	4
**Locations of work**	In-patient	12
	Out-patient	4
	Care home/residential homes	11
	Own homes	11
	Other	2

The theoretical basis of rehabilitation was explored for people with advanced dementia and three overarching themes were developed following analysis. The first explores how culture may affect the person with advanced dementia as well as the HCP working within a system or organisation. Knowledge was considered to be a key consideration when exploring the rehabilitation of people with advanced dementia, but this was closely linked to the personal values of the healthcare professional, which formed the final theme. Each theme will be explored in further detail and then the interaction between them considered.

Culture

Our participants discussed how there was a varied understanding of what "rehabilitation" means to people and how this differing understanding led to people with advanced dementia receiving a wide variety of interventions. This often related to one's understanding what the purpose of rehabilitation was from seeking to gain functional improvements to preventing decline. There was also discussion

For peer review only

about the consideration of how services and organisations' cultures affected the interventions that were delivered.

Meaning of "rehabilitation"

Determining what the term "rehabilitation" means was reported to be challenging for healthcare professionals generally. Our participants were very clear that for people with advanced dementia, tasks that were perceived to be 'simple' were actually very important and, although not deemed as "traditional rehabilitation" were actually important aspects of rehabilitation.

> *"we talk a lot in our training about the concept of rehab as what we find is if, if if they're not looking to get them back up on their feet, then that's not rehab. Whereas my argument that is actually for somebody with quite an advanced dementia, we have help them into a sitting position, can have massive functional benefits for them. So that in itself is rehab" (PT9)*

The term "traditional rehabilitation" was discussed by several of our participants and led back to the underlying biomedical principles that historically have been adopted by many healthcare professionals. This approach was focused on the injury or illness rather than the impairment that resulted from it.

> *"And I think that's possibly why we're missing the attitudes because we get to what you know all about anatomy and Physiology and this is how to treat a broken leg. This is how to treat a back pain. But they don't actually get taught how to embrace that full person centered approach to things." (PT7)*

There was also a noted discomfort that HCPs felt that they had to justify doing things differently – and thus outcome measures were described as being vital to "prove effectiveness" of their interventions. It was also discussed that often it was only the physical interventions that were perceived to be treatment – to such an extent that only the physical interventions were often documented. Our participants were very clear that these "non-traditional" approaches needed to be clearly documented to justify their interventions.

> *"I think is well, it's about how we document that because we don't always document those times when we've gone in and not been able to engage with somebody. We maybe say they weren't able to engage in our documentation, but we don't say we spent time engaging with that person and it resulted in an improved rapport, improved connection." (PT7)*

Complexity of patients was noted as requiring a different approach and people with advanced dementia were recognised as being highly complex and therefore needing care to be adapted accordingly.

> *"We're trying to pretend that we haven't got a complex system within which a complex patient is.... We're still, we're still trying to imagine that we've got a simple machine where if you put one thing in change, one of the dials, you're gonna get a completely different product out. And so it goes right back to*

For peer review only

undergraduate education and absolutely rethinking our model of physiotherapy." (PT1)

Determining the purpose

It was discussed that the focus of rehabilitation has often been to make a tangible improvement after an illness or injury which initially resulted in a decline in physical function. However, our participants questioned this and reported that often for people with advanced dementia, rehabilitation could be considered a slowing of the inevitable physical decline that is associated with the condition.

> *"….because it's a degenerative disorder and the, you know, the processes that these patients will deteriorate, I think it's, it's not just about improving things for them, but maintaining what they're able to do for as long as physically possible as well" (PT6)*

While there was a focus on preventing decline, there was also belief that people with advanced dementia can improve physically – therefore this should be a focus of the intervention. However, it was clear that this potential to improve physically required timely – and appropriately targeted – interventions, often before there was significant physical decline.

The unclear nature of interventions for people with advanced dementia makes it difficult to determine their trajectory of physical recovery, however, our HCPs felt very strongly that people needed to be given an opportunity to receive rehabilitation. Where the acute setting was often focused on discharging patients to ensure good patient "flow", giving these opportunities was challenging – although our participants were determined to offer as much as they possibly could, they recognised this was often insufficient.

> *"Yeah, time, time for us, I think is similar things is time how long you can spend with the patient. Obviously you know you have so many patients to split your time between. But our biggest time pressure is discharge pressures." (PT13)*

Impact of services/organisations

The influence of the service or organisation that a person was working within, or the person with advanced dementia was being treated by, had a significant impact on the interventions that could be delivered. The way that an organisation did, or in some cases did not, adapt their environments and policies to suit a person with advanced dementia would influence their journeys.

> *"The acute sector is massively, massively limited in what it can provide people with dementia. For for starters, the environment is completely wrong. It's wrong for people without dementia. It's completely wrong for people with dementia, so of course they're not gonna give their best selves." (PT4)*

For peer review only

While the acute setting had a significant focus on patient flow, the actual environment was deemed very unsuitable for people with advanced dementia. Noise, poor lighting and distractions would often add to challenging behaviours that the person might display.

> *"we do seem to have a bit of an issue, I call it beige bays, we create these spaces and we set people in them and we say now you stay there for six hours, 10 hours, 12 hours and only get up if you need to go to the toilet. We have a real kind of actively deprive people of activity during their stay and then get really shocked why they start getting really curious, slash bored out of their brains and wanting to get up and moving." (OT2)*

Organisations often were risk averse and fear of people falling and injuring themselves led to risk averse behaviours such as discouraging mobility or keeping people in bed, but HCPs feared this and actively tried to overcome these challenges. Discouraging activity led to deconditioning and deterioration of function which was considered a significant risk of harm in itself.

> *"I don't like the term deconditioning, because I think it's completely poor care and lack of facilitating to function… We've allowed this this appalling situation to happen where when our older people go into hospital, they deteriorate almost in front of in fact definitely in front of our eyes." (PT1)*

There was a frustration that often people with advanced dementia were restricted from undertaking "risky" activities, but there was little emphasis placed on how this would affect the person from an emotional perspective.

> *"I hear people talking about the talking about the risk of falling over the risk of pressure ulcers, that risk of infection. We have huge risks to personhood, to selfhood, to self esteem, to independence, to the ability to live independently." (PT1)*

Our HCPs also reported frustration with arbitrary timescales that patients were given to receive an intervention. Whether this was due to waiting lists for community services which were reported to be up to 40 weeks in some services, to the time limited services such as in-patient intermediate care or rehabilitation.

> *"….if you're gonna give people time, you need to look at the whole system, that of rehab, in those arbitrary. I mean, who came up with six weeks? I've got no idea. But that seems to be "if you can't get it and six weeks, then there's no point really" attitude." (PT9)*

Knowledge

Knowledge relating to dementia and a good understanding of person centred care was deemed vital to be able to effectively treat a person with advanced dementia. Where there was a lack of knowledge, there was often related poor outcomes for the person with advanced dementia.

Dementia

For peer review only

Knowledge relating to dementia was seen as being key to providing effective interventions for people with advanced dementia. Our HCPs described challenges in gaining this knowledge, with many relying on experience and learning "on the job". Undergraduate education for HCPs was universally felt to be inadequate and therefore, postgraduate education was vital, although difficult to access.

"The standard of university education on dementia for physios in particular and frailty is really, really poor and there's a lot and a lot of education that us more experienced physios need to be doing, but unfortunately. It doesn't really happen. And so I think that's one of the biggest challenges and that's why the words "no rehab potential" have have kind of sort of. They're still out there. Because there's not a lot of education around frailty and and older people" (PT4)

HCPs working in mental health settings found access to specific mental health training more accessible generally, although this was often generic rather than profession specific – this was particularly reported by physiotherapists who reported a lack of physiotherapy specific dementia training. There was a universal feeling amongst our participants that a large element of their role was about teaching and educating others – about dementia itself, but also the importance of delivering person centred care approaches to interventions. This related to other HCPs, but also to patients themselves and their carers.

A lack of knowledge and understanding was deemed to precipitate the belief that people with dementia had "no rehabilitation potential". Indeed, this term was reported to be used very commonly

"the staff who are labelling individuals of "no rehab potential" are often the less inexperienced staff ….. who's making a snapshot shot sort of decision within 10 minutes or five minutes." (PT2)

Person centred care

Person centred approaches to care were discussed in relation to a person with advanced dementia, but also in terms of how treatments should be adapted and personalised for everybody.

"It doesn't really matter if a patient has advanced dementia or if they're me, a 28 year old that's had knee surgery. If an exercise makes no sense to me, or if it's boring, or if it's not gonna help me get back to what I want to do, I'm probably not gonna do it. And. And really, if we make healthcare better for those with advanced dementia, we're gonna make it better for everyone, because what we're talking about there is personalized care, isn't it?" (PT4)

This emphasis on ensuring that interventions were tailored to individuals was reported to be even more important to a person with advanced dementia but understanding how to tailor interventions was variable and influenced by external factors such as time and availability of resources.

"…that time pressure because I think patients feel it and I think I might, you know, [allied health profession] colleagues feel it as well because literally as soon as people are either hitting a rehab ward or or getting into an [intermediate care]

For peer review only

bed, you're looking at discharge planning before you've even really got to the crooks of that rehab" (PT9)

Our HCPs reported that people were very aware of the concepts of person-centred approaches to care, but these were often seen as gold standard and their services were not always deemed able to achieve these standards. There was also suggestion that person centred care was not taught at undergraduate level in some professional groups, and this was something that had to be learnt as a post-graduate.

Personal values of the HCP

It was evident that the personal values of HCPs providing care for people with advanced dementia could significantly impact on the delivery of that care.

Curiosity

An interest in and taking time to get to know a person was deemed vital to be able to provide interventions to people with advanced dementia. However, a natural curiosity was evident amongst our participants. Where little formal education had been received, their curiosity to discover "what works" led them to a particular interest and passion for treating people with advanced dementia. However, this was deemed to be the exception rather than the rule and many of our participants reported feeling like a "*lone voice*" where few other people had an interest in this population.

"And the other point I just want to make is sometimes you feel like a lone voice out here kind of shouting about how important these things are. And I feel like I'm not on my own for a while." (PT7)

However, not all HCPs had this level of curiosity or interest about working with people with advanced dementia. Many of our participants provided teaching and education to others relating to dementia, but often with little positive engagement.

"I actually got onto the physio course for them for me to teach them and I did two consecutive years .....People weren't interested. There was this sort of, you know, and a room of 50 people just shut down, as if cognition and old and people with cognitive frailty was were something they'd never come across." (PT1)

This was particularly reported amongst physiotherapists and there was a belief that occupational therapists were more involved in treating people with dementia or cognitive difficulties historically, although there was a feeling that this attitude was beginning to change. There was further evidence to support this with our participants discussing the role of physiotherapists in mental health teams being much less advanced than the role of occupational therapists.

"I was one of the first physiotherapist who was hired to work in a nursing home and in [country], and I've been, I've had a uh, I've worked a lot with nurses and caregivers and they've been really interested in what what I could offer. I have [with] the physiotherapist. They weren't interested." (PT5)

For peer review only

Optimism/wisdom

There was considerable optimism amongst our participants that people with advanced dementia could effectively receive rehabilitation and other interventions, but this came from positive previous experience – thus their wisdom. It was notable that sharing this experience and positive outcomes was used as a way to engage other HCPs in treating people with advanced dementia.

Knowledge of negative outcomes for people with dementia also led HCPs to be more proactive in their approaches, often actively seeking out patients who would benefit from specialist interventions.

> *"And so so from now on, we've also changed our practice. Whenever anyone go to the to that trust, we've phone and we make sure and we may actually and we we go out and we had somebody….. So I do think we don't want to miss that opportunity." (PT10)*

However, this optimism was often diminished by challenges relating to service pressures and limitation that the HCPs had to work within. Unsuitable ward environments were a particular challenge and the HCPs accepted the fact that the person with advanced dementia was unlikely to be able to improve in that setting and therefore focused their attention on ensuring they could be discharged to the most appropriate location to facilitate and rehabilitation. Where this optimism was reduced, HCPs noted feeling very alone and isolated when trying to "fight" for people with dementia.

Compassion

It was evident that our participants felt a significant amount of compassion towards their patients, but particularly those with advanced dementia. There was a belief that people with advanced dementia were often stigmatised and labelled which prevented them from receiving the care that they needed and deserved.

> *"…there's this been this historic thing about "no rehab potential". And the consistent negative labelling of our people that we work with, I think of our people as being made invisible by so many post, it notes saying stuck upon their person saying. "No capacity, no rehab potential behaviours that challenge aggressive." That that's always been the negative, negative and pejorative labelling." (PT1)*

There was also a feeling of guilt and frustration where a HCP was not able to provide the intervention they wanted to – usually due to resource pressures or the culture of the organisation. However, it was evident that the individuals attitude was key and their beliefs and engagement with people with advanced dementia had a pivotal role in determining what intervention was delivered.

> *"attitude is huge. I think I completely agree with that. I think if you if you don't have the right attitude, nothing really follows on and I get flow from that and this sort of three words that stand out for me are time, connection and value. So value*

For peer review only

in the person value in the carer value in their journey and where they've got to until now." (PT7)

Discussion

The aims of the study were to explore the theoretical basis of providing and delivering rehabilitation for people with advanced dementia. Our participants described the key aspects to consider the culture of rehabilitation and raised questions about what this means, but also what other people and HCPs perceive it to be. There was a common belief that knowledge was key and where there was a lack of knowledge about treating people with advanced dementia outcomes were poor – or people simply weren't offered interventions. However, there was a noted importance on the values of the HCP themselves and how these values could be directly influenced by the culture of the organisation and the knowledge about how best to manage people with advanced dementia.

Our data suggests an important relationship between these factors and for a person with advanced dementia to receive optimal interventions, there needs to be a symbiotic relationship between all three factors (Table 1). Where any one of the elements is missing, the person with dementia will not receive the care that they need.

Our data suggests that knowledge is a key domain to ensure people with advanced dementia receive optimal care, however it was clear that lack of education relating to dementia and person-centred care approaches would significantly affect interventions. Lack of knowledge amongst healthcare professionals is inconsistent with one large study undertaken by Alzheimer's Disease International suggesting that 62% of healthcare professionals believed that dementia is a natural part of ageing and supported other studies findings stating the belief there was nothing that could be done to manage it [15, 16]. However, it is unclear whether and how this relates to specific professions. Our data suggests a lack of undergraduate training for physiotherapists relating to dementia, however a Canadian study surveyed physiotherapists and found that levels of knowledge relating to dementia was high, but cited lack of confidence to manage behavioural and cognitive difficulties [17]. Interestingly, similar research exploring the attitudes of physiotherapy students found that 53% felt their academic training was sufficient to equip them with the skills to work with people with dementia [18], suggesting a reduction in confidence from undergraduate level to postgraduate level.

It was very evident from our data that the personal values, and thus the attitude, towards a person with advanced dementia was pivotal in determining the care that was delivered and received. Our participants were all experts in treating people with advanced dementia and therefore had compassion and interest in treating this population, however they reported numerous examples where poor personal values of the HCP negatively affected the person with dementia – and thus their outcomes. Research has explored the importance of providing education and training for healthcare professionals relating to dementia in the acute setting [19], with a highlighted requisite that this education supports the development of empathy and understanding relating to the person with dementia [19, 20]. However, much of this research focuses on attitudes in relation to knowledge rather than the person values of the HCP and how these influence the care provided.

The culture of the organisation was suggested to influence the HCPs values and conversely, HCPs with poor values relating to dementia could negatively affect the culture of the organisation. The

For peer review only

culture of care has been linked to the value placed on the person with dementia [21], thus placing greater value on the person facilitates a culture which supports them. However, it has been suggested that education alone is not sufficient to facilitate this positive culture [19, 20].

Our data suggests that the inter-relationship between the knowledge and values of the HCP and the culture of the organisation are all key to successful outcomes. Our theoretical model (Table 2) suggests that only positive attributes of all three domains will result in successful outcomes for people with dementia.

Table 2 - detailing the interaction of factors and the outcomes for the person with dementia and the HCP

Values of the HCP	Culture of the organisation	Knowledge of the HCP	Outcomes	
			For the person living with dementia	For the HCP
Good	Good	Good	Positive outcomes	Satisfaction and ability to make a positive improvement in care
Good	Good	Poor	Poor outcomes	Willingness and desire to improve outcomes, but insufficient knowledge to achieve good outcomes
Good	Poor	Poor	Poor outcomes	Frustration that the HCP values the person with dementia but has neither the positive culture within the organisation or the knowledge to make a positive change.
Good	Poor	Good	Poor outcomes	Frustration of HCP being unable to achieve good outcomes for the patient as the organisation does not support their interventions
Poor	Poor	Good	Poor outcomes	Unable to use the knowledge they have in an effective manner
Poor	Poor	Poor	Vicious spiral of worsening outcomes	Lack of interest or concern from HCP. Skills and

				knowledge deteriorate further.

Conclusion

This qualitative study contributes to the knowledge about key factors when providing rehabilitation interventions for people with advanced dementia. It can be used to inform future implementation of dementia-specific interventions as it is a necessity to optimise knowledge, personal values and the organisational culture in order for outcomes to be effective. It further adds to the evidence base that people with advanced dementia need to be provided with the right environment and opportunities to benefit from rehabilitation interventions.

For peer review only

Author Contributions

Conceptualization: AH.; methodology: AH, FM, VG; formal analysis: AH, FM, VG. ; data curation; AH, FM, VG..; writing—original draft: AH.; writing—review and editing: AH, FM, VG.; supervision: VG.; All authors have read and agreed to the published version of the manuscript.

Funding

This study is independent research funded by the National Institute for Health and Care Research (NIHR) School for Primary Care Research (119247) and supported by the NIHR Applied Research Collaboration South West Peninsula. The views expressed in this publication are those of the author(s) and not necessarily those of the National Institute for Health Research or the Department of Health and Social Care.

Institutional Review Board Statement

The study was conducted according to the guidelines of the Declaration of Helsinki, and approved by the and ethical approval was obtained from the University of Exeter’s College of Medicine and Health Research Ethics Committee (reference 515745).

Informed Consent Statement

Informed consent was obtained from all subjects involved in the study.

Data Availability Statement

The datasets generated during and/or analyzed during the current study are available from the corresponding author upon reasonable request.

Acknowledgments

The authors thank all the participants who devoted their time to participate in this study.

Conflicts of Interest

The authors declare no conflict of interest.

For peer review only

References

1. Organization, W.H., *WHO global strategy on people-centred and integrated health services: interim report*. 2015, World Health Organization.
2. Organization, W.H., *Dementia: a public health priority*. 2012: World Health Organization.
3. Ahmadi-Abhari, S., et al., *Temporal trend in dementia incidence since 2002 and projections for prevalence in England and Wales to 2040: modelling study.* bmj, 2017. **358**.
4. Mitchell, S.L., *Advanced dementia.* New England Journal of Medicine, 2015. **372**(26): p. 2533-2540.
5. Banerjee, S., et al., *Quality of life in dementia: more than just cognition. An analysis of associations with quality of life in dementia.* Journal of Neurology, Neurosurgery & Psychiatry, 2006. **77**(2): p. 146-148.
6. Hall, A.J., S. Febrey, and V.A. Goodwin, *Physical interventions for people with more advanced dementia–a scoping review.* BMC geriatrics, 2021. **21**(1): p. 1-10.
7. Forbes, D., et al., *Physical activity programs for persons with dementia.* Cochrane Database of Systematic Reviews, 2008(3).
8. Lamb, S., et al., *Aerobic and strength training exercise programme for cognitive impairment in people with mild to moderate dementia: the DAPA RCT.* Health Technology Assessment, 2018. **22**(28).
9. Blondell, S.J., R. Hammersley-Mather, and J.L. Veerman, *Does physical activity prevent cognitive decline and dementia?: A systematic review and meta-analysis of longitudinal studies.* BMC public health, 2014. **14**(1): p. 1-12.
10. Groot, C., et al., *The effect of physical activity on cognitive function in patients with dementia: a meta-analysis of randomized control trials.* Ageing research reviews, 2016. **25**: p. 13-23.

11. Cameron, I.D., *Models of rehabilitation–commonalities of interventions that work and of those that do not.* Disability and rehabilitation, 2010. **32**(12): p. 1051-1058.
12. Leplège, A., C. Barral, and K. McPherson, *Conceptualizing disability to inform rehabilitation: Historical and epistemological perspectives*, in *Rethinking Rehabilitation*. 2015, CRC Press. p. 42-65.
13. Jorgensen, P., *Concepts of body and health in physiotherapy: The meaning of the social/cultural aspects of life.* Physiotherapy theory and practice, 2000. **16**(2): p. 105-115.
14. Clarke, V. and V. Braun, *Thematic analysis*, in *Encyclopedia of critical psychology*. 2014, Springer. p. 1947-1952.
15. Lynch, C., *World Alzheimer Report 2019: Attitudes to dementia, a global survey: Public health: Engaging people in ADRD research.* Alzheimer's & Dementia, 2020. **16**: p. e038255.
16. Zhao, W., et al., *Hospital healthcare professionals' knowledge of dementia and attitudes towards dementia care: A cross-sectional study.* Journal of Clinical Nursing, 2022. **31**(13-14): p. 1786-1799.
17. Hunter, S.W. and A. Divine, *Understanding the factors influencing physiotherapists' attitudes towards working with people living with dementia.* Physiotherapy Theory and Practice, 2021. **37**(12): p. 1448-1455.
18. Hunter, S.W., et al., *Physiotherapy students' attitudes toward working with people with dementia: A cross-sectional survey.* Physical & Occupational Therapy In Geriatrics, 2020. **38**(1): p. 56-66.
19. Cowdell, F., *Care of older people with dementia in an acute hospital setting.* Nursing Standard (through 2013), 2010. **24**(23): p. 42.
20. Moyle, W., et al., *Care staff attitudes and experiences of working with older people with dementia.* Australasian Journal on Ageing, 2011. **30**(4): p. 186-190.
21. Houghton, C., et al., *Healthcare staffs' experiences and perceptions of caring for people with dementia in the acute setting: Qualitative evidence synthesis.* International Journal of Nursing Studies, 2016. **61**: p. 104-116.

For peer review only

COREQ (COnsolidated criteria for REporting Qualitative research) Checklist

A checklist of items that should be included in reports of qualitative research. You must report the page number in your manuscript where you consider each of the items listed in this checklist. If you have not included this information, either revise your manuscript accordingly before submitting or note N/A.

Topic	Item No.	Guide Questions/Description	Reported on Page No.
**Domain 1: Research team and reflexivity**			
Personal characteristics			
Interviewer/facilitator	1	Which author/s conducted the interview or focus group?	
Credentials	2	What were the researcher's credentials? E.g. PhD, MD	
Occupation	3	What was their occupation at the time of the study?	
Gender	4	Was the researcher male or female?	
Experience and training	5	What experience or training did the researcher have?	
Relationship with participants			
Relationship established	6	Was a relationship established prior to study commencement?	
Participant knowledge of the interviewer	7	What did the participants know about the researcher? e.g. personal goals, reasons for doing the research	
Interviewer characteristics	8	What characteristics were reported about the inter viewer/facilitator? e.g. Bias, assumptions, reasons and interests in the research topic	
**Domain 2: Study design**			
Theoretical framework			
Methodological orientation and Theory	9	What methodological orientation was stated to underpin the study? e.g. grounded theory, discourse analysis, ethnography, phenomenology, content analysis	
Participant selection			
Sampling	10	How were participants selected? e.g. purposive, convenience, consecutive, snowball	
Method of approach	11	How were participants approached? e.g. face-to-face, telephone, mail, email	
Sample size	12	How many participants were in the study?	
Non-participation	13	How many people refused to participate or dropped out? Reasons?	
Setting			
Setting of data collection	14	Where was the data collected? e.g. home, clinic, workplace	
Presence of non-participants	15	Was anyone else present besides the participants and researchers?	
Description of sample	16	What are the important characteristics of the sample? e.g. demographic data, date	
Data collection			
Interview guide	17	Were questions, prompts, guides provided by the authors? Was it pilot tested?	
Repeat interviews	18	Were repeat inter views carried out? If yes, how many?	
Audio/visual recording	19	Did the research use audio or visual recording to collect the data?	
Field notes	20	Were field notes made during and/or after the inter view or focus group?	
Duration	21	What was the duration of the inter views or focus group?	
Data saturation	22	Was data saturation discussed?	
Transcripts returned	23	Were transcripts returned to participants for comment and/or	

For peer review only

Topic	Item No.	Guide Questions/Description	Reported on Page No.
		correction?	
**Domain 3: analysis and findings**			
Data analysis			
Number of data coders	24	How many data coders coded the data?	
Description of the coding tree	25	Did authors provide a description of the coding tree?	
Derivation of themes	26	Were themes identified in advance or derived from the data?	
Software	27	What software, if applicable, was used to manage the data?	
Participant checking	28	Did participants provide feedback on the findings?	
Reporting			
Quotations presented	29	Were participant quotations presented to illustrate the themes/findings? Was each quotation identified? e.g. participant number	
Data and findings consistent	30	Was there consistency between the data presented and the findings?	
Clarity of major themes	31	Were major themes clearly presented in the findings?	
Clarity of minor themes	32	Is there a description of diverse cases or discussion of minor themes?	

Developed from: Tong A, Sainsbury P, Craig J. Consolidated criteria for reporting qualitative research (COREQ): a 32-item checklist for interviews and focus groups. *International Journal for Quality in Health Care*. 2007. Volume 19, Number 6: pp. 349 – 357

Once you have completed this checklist, please save a copy and upload it as part of your submission. DO NOT include this checklist as part of the main manuscript document. It must be uploaded as a separate file.

For peer review only

BMJ Open

A qualitative study exploring health care professionals perceptions of providing rehabilitation for people with advanced dementia

Journal:	*BMJ Open*
Manuscript ID	bmjopen-2023-072432.R1
Article Type:	Original research
Date Submitted by the Author:	09-May-2023
Complete List of Authors:	Hall, Abigail J.; University of Exeter<br>Manning, Fay; University of Exeter Medical School, Exeter Medical School<br>Goodwin, Victoria; University of Exeter, PenCLAHRC
**Primary Subject Heading**:	Rehabilitation medicine
Secondary Subject Heading:	Qualitative research
Keywords:	Dementia, REHABILITATION MEDICINE, QUALITATIVE RESEARCH

SCHOLARONE™
Manuscripts

I, the Submitting Author has the right to grant and does grant on behalf of all authors of the Work (as defined in the below author licence), an exclusive licence and/or a non-exclusive licence for contributions from authors who are: i) UK Crown employees; ii) where BMJ has agreed a CC-BY licence shall apply, and/or iii) in accordance with the terms applicable for US Federal Government officers or employees acting as part of their official duties; on a worldwide, perpetual, irrevocable, royalty-free basis to BMJ Publishing Group Ltd ("BMJ") its licensees and where the relevant Journal is co-owned by BMJ to the co-owners of the Journal, to publish the Work in this journal and any other BMJ products and to exploit all rights, as set out in our licence.

The Submitting Author accepts and understands that any supply made under these terms is made by BMJ to the Submitting Author unless you are acting as an employee on behalf of your employer or a postgraduate student of an affiliated institution which is paying any applicable article publishing charge ("APC") for Open Access articles. Where the Submitting Author wishes to make the Work available on an Open Access basis (and intends to pay the relevant APC), the terms of reuse of such Open Access shall be governed by a Creative Commons licence – details of these licences and which Creative Commons licence will apply to this Work are set out in our licence referred to above.

Other than as permitted in any relevant BMJ Author's Self Archiving Policies, I confirm this Work has not been accepted for publication elsewhere, is not being considered for publication elsewhere and does not duplicate material already published. I confirm all authors consent to publication of this Work and authorise the granting of this licence.

For peer review only

A qualitative study exploring health care professionals perceptions of providing rehabilitation for people with advanced dementia

Abigail J Hall (corresponding author) BSc, MSc, PhD, MCSP

University of Exeter, Heavitree Road, Exeter, EX1 2LU

a.hall4@exeter.ac.uk

Fay Manning BSc, MSc, PhD

University of Exeter, Heavitree Road, Exeter, EX1 2LU

Victoria A Goodwin, MBE, BSc, MSc, PhD, FCSP,

University of Exeter, Heavitree Road, Exeter, EX1 2LU

For peer review only

Abstract

Objectives

The aims of the study were to explore health care professionals rehabilitation principles for providing and delivering rehabilitation interventions for people with advanced dementia.

Design

This was a qualitative study with three focus groups undertaken virtually. The data was analysed using a process of reflexive thematic analysis in order to gain an in depth understanding of rehabilitation principles for this population.

Setting and Participants

20 healthcare professionals who were experts in treating and rehabilitating people with advanced dementia were recruited. These healthcare professionals had a wide range of experience in a variety of different settings including primary care, secondary care as well as specialist mental health teams. Purposive sampling focused on the requirement for participants to have significant experience of treating people with dementia.

Results

Three overarching themes were developed following analysis. The first explores how the culture of an organisation affects a person with advanced dementia as well as the healthcare professional. The organisation needed to promote positive approaches to person centred care and provide effective situational leadership to embed such approaches. Knowledge was a key consideration and was closely linked to the personal values of the healthcare professional, which formed the final theme. This study suggests that the interrelationship of these three factors influenced the outcomes for the person with dementia and effective outcomes required effective consideration of all domains.

Conclusions

Effective interventions for people with advanced dementia require the healthcare professional to have the knowledge and personal values to support the person, but the culture of the organisation is also key to ensure that the healthcare professional is able to demonstrate these personal values and knowledge effectively.

For peer review only

Article Summary

Strengths and limitations

- We recognise that this was a small-scale qualitative study with a limited range of healthcare professionals taking part, however, sampling with deliberately specific and engaged HCPs who were specialists in treating people with very advanced dementia – of which there is a limited sample of people due to the specialist nature of interventions.
- The methods of data collection and analysis were robust and followed clear guidance for undertaking such qualitative work.
- The study was reported using COREQ (see supplementary material) to ensure clear and transparent methods and analysis.

Background

Dementia is a neurological disorder resulting in physical and cognitive decline, found typically in older adults [1, 2]. It is estimated that by 2040, there will be 1.2 million people with dementia living in the UK – a 57% increase from 2016 [3], suggesting that dementia will become a national – and global – challenge to healthcare services. Advanced dementia is characterised by profound communication, memory and physical disturbances [4], which often result in significant impairments in a person's ability to undertake activities of daily living and a perceived decline in quality of life [5].

A variety of healthcare professionals (HCPs) can be involved in trying to reduce the physical declines associated with advanced dementia, including physiotherapists and occupational therapists. However, there remains little evidence to support physical interventions for people with dementia [6-10]. This lack of evidence is particularly evident for people with more severe dementia, where physical interventions to improve physical ability – or reduce the speed of decline – are largely not understood [6].

There is much debate about the approach that is suitable for people with advanced dementia, with greater emphasis being placed on adopting a biopsychosocial model, where functioning and participation is key. Some 60 years ago, a key paper - "The need for a new model: A challenge for biomedicine" [11] argued that the dominant biomedical model of disease was insufficient for a complete understanding of health. He stated that a biomedical approach ''*assumes disease to be fully accounted for by deviations from the norm of measurable, biological variables. It leaves no room within its framework for the social, psychological and behavioural dimensions of illness*'' (p. 130). Thus, the importance of merging biomedical and psychosocial aspects of health, which encouraged clinicians to consider not only biological components, but also the individual and societal contexts of the individual's experiences. However, the challenge that this poses to healthcare professionals is poorly understood – especially for people living with more advanced dementia.

For peer review only

The experience of a variety of health care professionals treating people with dementia has been explored in a variety of settings [12, 13], including residential aged care facilities [13, 14] and acute settings [15]. A recent review exploring the experiences of caring for people with dementia in acute settings has highlighted the importance of developing knowledge of person centred approaches to care, however research has also highlighted a lack of understanding about the importance of the environment and how this may impact care [16]. However, evidence suggests that providing rehabilitation interventions for people with more advanced dementia is different to those with more mild forms [6, 17]. Thus, the aims of the study were to explore the rehabilitation principles of HCPs providing and delivering rehabilitation interventions for people with advanced dementia in a variety of settings including acute, community and residential care.

Methods/design

During August and September 2022, three semi-structured focus groups were undertaken with a range of HCPs. These health professionals were all experts in the management or rehabilitation of people living with dementia. The focus groups were undertaken virtually to reduce barriers due to geographical limitations and enabled international experts to contribute. The study has been reported according to the COREQ guidelines ((see supplementary material).

Setting and participants

A purposive sampling strategy was employed to seek HCPs who specialise in working with people with advanced dementia. A targeted social media advert sought professionals with advanced skills working with people with advanced dementia and pre-existing networks were drawn upon to recruit participants. Some of these participants were known to the researchers in a professional capacity due to previous work undertaken in this area. This did not affect the data collection.

Inclusion criteria;

- Specialist working with people with dementia as a physiotherapist, occupational therapist, psychologist, nurse, or other related profession. (“Specialist” refers to the level of experience and training that a HCP has and an expectation that they working in an advanced role relating to the care of people with dementia)

Participants who responded to the social media advert or initial email were sent a follow up email with the participant information sheet attached. At this point they were offered an opportunity to discuss the study further if they had any particular questions. If they were happy to take part in the study, they were asked to provide informed consent prior to the commencement of the focus group. Participants were informed that they were under no obligation to participate in the focus group and they may withdraw from it at any time, without any negative consequence, up until the point where the data were fully anonymised.

Provided the participant met the inclusion criteria, all participants who consented to take part were invited to join a focus group. Recruitment continued until sufficient data was obtained to be able to answer the aim of the study and no new data was being generated.

For peer review only

Data collection

The focus groups aimed to explore the challenges and techniques required to effectively manage people with more advanced dementia, while also considering the principles of rehabilitation for this population. The focus groups were facilitated by two experienced qualitative researchers (AH and FM). FM acted as a 'second facilitator/observer', taking notes and observations pertaining to interaction of the group. Both researchers have undertaken training courses pertaining to qualitative analysis and have published multiple qualitative papers.

Microsoft Teams was used to undertake the focus groups and they were recorded and automatically transcribed. The transcription function has been used by the researcher and found to be accurate, although the transcripts were all checked for accuracy. The focus groups lasted for a maximum of 90 minutes and included time for participants to introduce themselves and their roles to other members of the group. The participants were informed that this study formed part of a larger piece of work aiming to develop interventions for people with advanced dementia. A semi-structured guide (see supplementary material) was used to guide the conversation of the focus groups. The topic guide was not piloted prior to the initial focus group as it was anticipated that it could be adapted for subsequent focus groups if needed. This was not required and the same topic guide was used for all interviews. The data was anonymised so that individual participants could not be identified, and all participants consented to their data being transcribed and analysed. Transcripts were not reviewed by participants. The data collection from the focus groups provided rich and detailed data and no further follow-up interviews were deemed necessary although people were offered the opportunity to discuss any further points following completion of the focus group if there were points they had not been able to raise during the meeting.

Patient and Public Involvement (PPI)

A patient and public involvement (PPI) group was developed to help inform the research undertaken during this study and as part of a wider programme of work. The group has been evolving and developing and now consists of people with a variety of experiences of dementia. The group includes people living with dementia, carers of people with dementia and two health care professional who have personal experience of dementia.

Initial discussions focused on the challenges they had experienced with getting rehabilitation for people who have dementia – especially when the person had advanced dementia or was living in a care home. Several themes generated from this discussion formed part of the focus group discussion. Due to practical challenges, none of the PPI group were able to participate in the focus groups, but discussion was held with representatives following the groups and helped guide the analysis and results.

Analysis

For peer review only

Analysis commenced after the first focus group was completed and continued through the following focus groups. As the data collection and initial analysis ran simultaneously arising themes or gaps could be probed accordingly in the remaining focus groups.

NVivo 11 (QSR International) was used to organize and code the data and facilitated a process of reflexive thematic analysis to be undertaken as suggested by Braun and Clarke [18]. Familiarisation of the data was undertaken be reading and re-reading the transcripts. A process of systematic open coding was then commenced and initial ideas for themes were noted. Following this open coding, initial themes were generated, discussed and explored with the wider research team. These discussions lead to further refinement of the themes, and definitions of the themes to be written to ensure clarity between the research team. For example, there were multiple codes relating to participants using positive success stories to educate others, this was collated into a minor theme of "optimism/wisdom" which was then classified within the "personal values of the HCP". This process was repeated for all the data until themes were developed. This process was an iterative process and resulted in several refinements of named themes. These themes were then discussed with a member of the patient and public involvement and engagement (PPIE) group who were supporting and collaborating on the overarching project.

Ethics

Ethical approval was obtained from the University of Exeter's College of Medicine and Health Research Ethics Committee (reference 515745).

Findings

20 healthcare professionals (HCPs) took part in three focus groups (Table 1). Only one person who had initially expressed an interest in taking part did not reply to the meeting invite and therefore did not take part. Following the third focus group, it was felt that no new themes were occurring and therefore it was believed that theoretical data saturation had been reached and no further focus groups were planned. There were no withdrawals from the study at any stages. The HCPs reported an average of eighteen years of experience and an average of thirteen years specializing specifically with people with dementia. The settings of their experience were broad, including in-patient settings, out-patient clinics, patients' own homes and residential/nursing settings.

Table 1 - characteristics of participants (n=20)

**Sex**	**Female**	**19**
	Male	1
**Profession**	Physiotherapist	14
	Occupational Therapist	3
	Nurse	2
	Psychologist	1
**NHS Grade / Banding**	6	6
	7	8
	8+	2

For peer review only

	N/A	4
**Years of clinical experience**	0-5	1
	6 - 10	2
	11-15	2
	16 - 20	3
	21 - 25	6
	26 - 30	2
	30+	3
	Not reported	1
**Years of experience working with people with advanced dementia**		
	0-5	4
	6 - 10	2
	11-15	6
	16 - 20	5
	21 - 25	2
	26 - 30	1
	30+	0
**Location**	England	13
	Wales	2
	Scotland	4
	Denmark	1
**% of caseload of people with dementia**		
	0 - 25	1
	26 - 50	1
	51 - 75	6
	76 - 100	8
	Not currently working clinically	4
**Locations of work**	In-patient	12
	Out-patient	4
	Care home/residential homes	11
	Own homes	11
	Other	2

The principles of rehabilitation was explored for people with advanced dementia and three overarching themes were developed following analysis. The first explores how culture may affect the person with advanced dementia as well as the HCP working within a system or organisation. Knowledge was considered to be a key consideration when exploring the rehabilitation of people with advanced dementia, but this was closely linked to the personal values of the healthcare professional, which formed the final theme. Each theme will be explored in further detail and then the interaction between them considered.

Organisational Culture

Our participants discussed how there was a varied understanding of what “rehabilitation” means to people and how this differing understanding led to people with advanced dementia receiving a wide variety of interventions. This often related to one’s understanding what the purpose of rehabilitation was from seeking to gain functional improvements to preventing decline. There was also discussion about the consideration of how services and organisations’ cultures affected the interventions that were delivered.

Determining what the term “rehabilitation” means was reported to be challenging for healthcare professionals generally. Our participants were very clear that for people with advanced dementia, tasks that were perceived to be ‘simple’ were actually very important and, although not deemed as “traditional rehabilitation” were actually important aspects of rehabilitation.

> *“we talk a lot in our training about the concept of rehab as what we find is if…. they're not looking to get them back up on their feet, then that's not rehab. Whereas my argument that is actually for somebody with quite an advanced dementia, we have help them into a sitting position, can have massive functional benefits for them. So that in itself is rehab” (PT9)*

The term “traditional rehabilitation” was discussed in all focus groups and led back to the underlying biomedical principles that historically have been adopted by many healthcare professionals. This approach was focused on the injury or illness rather than the impairment that resulted from it.

> *“…..because we get to what you know all about anatomy and physiology and this is how to treat a broken leg. This is how to treat a back pain. But they don't actually get taught how to embrace that full person centered approach to things.” (PT7)*

There was also a noted discomfort that HCPs felt that they had to justify doing things differently – and thus outcome measures were described as being vital to “prove effectiveness” of their interventions. It was also discussed that often it was only the physical interventions that were perceived to be treatment – to such an extent that only the physical interventions were often documented. Our participants were very clear that these “non-traditional” approaches needed to be clearly documented to justify their interventions.

> *“I think is well, it's about how we document that because we don't always document those times when we've gone in and not been able to engage with somebody. We maybe say they weren't able to engage in our documentation, but we don't say we spent time engaging with that person and it resulted in an improved rapport, improved connection.” (PT7)*

Complexity of patients was noted as requiring a different approach and people with advanced dementia were recognised as being highly complex and therefore needing care to be adapted accordingly.

> *“We're trying to pretend that we haven't got a complex system….which a complex patient is…. we're still trying to imagine that we've got a simple machine where if you put one thing in change, one of the dials, you're gonna get a completely*

For peer review only

different product out. And so it goes right back to undergraduate education and absolutely rethinking our model of physiotherapy." (PT1)

It was discussed that the focus of rehabilitation has often been to make a tangible improvement after an illness or injury which initially resulted in a decline in physical function. However, our participants questioned this and reported that often for people with advanced dementia, rehabilitation could be considered a slowing of the inevitable physical decline that is associated with the condition.

"….because it's a degenerative disorder and …. the processes that these patients will deteriorate…..it's not just about improving things for them, but maintaining what they're able to do for as long as physically possible as well" (PT6)

While there was a focus on preventing decline, there was also belief that people with advanced dementia can improve physically – therefore this should be a focus of the intervention. However, it was clear that this potential to improve physically required timely – and appropriately targeted – interventions, often before there was significant physical decline.

The unclear nature of interventions for people with advanced dementia makes it difficult to determine their trajectory of physical recovery, however, our HCPs felt very strongly that people needed to be given an opportunity to receive rehabilitation. In the acute setting, giving these opportunities was challenging due to the focus on discharging patients to ensure good patient "flow". Despite this challenge, our participants were determined to offer as much as they possibly could, they recognised this was often insufficient.

"Yeah, time, time for us… how long you can spend with the patient. Obviously you know you have so many patients to split your time between. But our biggest time pressure is discharge pressures." (PT13)

The influence of the service or organisation that a person was working within had a significant impact on the interventions that could be delivered. The way that an organisation did, or in some cases did not, adapt their environments and policies to suit a person with advanced dementia would influence their journeys.

"The acute sector is massively, massively limited in what it can provide people with dementia…. the environment is completely wrong. It's wrong for people without dementia. It's completely wrong for people with dementia, so of course they're not gonna give their best selves." (PT4)

While the acute setting had a significant focus on patient flow, the actual environment was deemed very unsuitable for people with advanced dementia. Noise, inappropriate lighting and distractions would often add to challenging behaviours that the person might display.

"we do seem to have a bit of an issue, I call it beige bays, we create these spaces and we set people in them and we say 'now you stay there for six hours, 10 hours, 12 hours and only get up if you need to go to the toilet'. We….actively deprive people of activity during their stay and then get really shocked why they start

For peer review only

getting really curious/bored out of their brains and wanting to get up and moving." (OT2)

Organisations often were risk averse and fear of people falling and injuring themselves led to risk averse behaviours such as discouraging mobility or keeping people in bed, but HCPs feared this and actively tried to overcome these challenges. Discouraging activity led to deconditioning and deterioration of function which was considered a significant risk of harm in itself.

"I don't like the term deconditioning, because I think it's completely poor care and lack of facilitating to function… We've allowed this this appalling situation to happen where when our older people go into hospital, they deteriorate almost in front of in fact definitely in front of our eyes." (PT1)

There was a frustration that often people with advanced dementia were restricted from undertaking "risky" activities, but there was little emphasis placed on how this would affect the person from an emotional perspective.

"I hear people talking about the talking about the risk of falling over the risk of pressure ulcers, that risk of infection. We have huge risks to personhood, to selfhood, to self esteem, to independence, to the ability to live independently." (PT1)

Our HCPs also reported frustration with arbitrary timescales that patients were given to receive an intervention. Whether this was due to waiting lists for community services which were reported to be up to 40 weeks in some services, to the time limited services such as in-patient intermediate care or rehabilitation.

"….if you're gonna give people time, you need to look at the whole system, that of rehab….who came up with six weeks? I've got no idea. But that seems to be "if you can't get it and six weeks, then there's no point really" attitude." (PT9)

Knowledge

Knowledge relating to dementia and a good understanding of person centred care was deemed vital to be able to effectively treat a person with advanced dementia. Where there was a lack of knowledge, there was often related poor outcomes for the person with advanced dementia.

Knowledge relating to dementia was seen as being key to providing effective interventions for people with advanced dementia. Our HCPs described challenges in gaining this knowledge, with many relying on experience and learning "on the job". Undergraduate education for HCPs was universally felt to be inadequate and therefore, postgraduate education was vital, although difficult to access.

"The standard of university education on dementia for physios in particular and frailty is really, really poor and there's a lot and a lot of education that us more experienced physios need to be doing…. and that's why the words "no rehab potential" ….[are] still out there. Because there's not a lot of education around frailty and older people" (PT4)

For peer review only

HCPs working in mental health settings found access to specific mental health training more accessible generally, although this was often generic rather than profession specific – this was particularly reported by physiotherapists who reported a lack of physiotherapy specific dementia training. There was a universal feeling amongst our participants that a large element of their role was about teaching and educating others – about dementia itself, but also the importance of delivering person centred care approaches to interventions. This related to other HCPs, but also to patients themselves and their carers.

A lack of knowledge and understanding was deemed to precipitate the belief that people with dementia had "no rehabilitation potential". Indeed, this term was reported to be used very commonly.

"the staff who are labelling individuals of "no rehab potential" are often the less inexperienced staff ….. making a snapshot sort of decision within 10 minutes or five minutes." (PT2)

Person centred approaches to care were discussed in relation to a person with advanced dementia, but also in terms of how treatments should be adapted and personalised for everybody.

"It doesn't really matter if a patient has advanced dementia or if they're me, a 28 year old that's had knee surgery. If an exercise makes no sense to me, or if it's boring, or if it's not gonna help me get back to what I want to do, I'm probably not gonna do it. And really, if we make healthcare better for those with advanced dementia, we're gonna make it better for everyone, because what we're talking about there is personalized care, isn't it?" (PT4)

This emphasis on ensuring that interventions were tailored to individuals was reported to be even more important to a person with advanced dementia but understanding how to tailor interventions was variable and influenced by external factors such as time and availability of resources.

"…that time pressure because I think patients feel it and I think I might, you know, [allied health profession] colleagues feel it as well because literally as soon as people are either hitting a rehab ward or getting into an [intermediate care] bed, you're looking at discharge planning before you've even really got to the crux of that rehab" (PT9)

Our HCPs reported that people were very aware of the concepts of person-centred approaches to care, but these were often seen as gold standard and their services were not always deemed able to achieve these standards. There was also suggestion that person centred care was not taught at undergraduate level in some professional groups, and this was something that had to be learnt as a post-graduate.

Personal values of the HCP

It was evident that the personal values of HCPs providing care for people with advanced dementia could significantly impact on the delivery of that care.

For peer review only

An interest in and taking time to get to know a person was deemed vital to be able to provide interventions to people with advanced dementia. However, a natural curiosity was evident amongst our participants. Where little formal education had been received, their curiosity to discover "what works" led them to a particular interest and passion for treating people with advanced dementia. However, this was deemed to be the exception rather than the rule and many of our participants reported feeling like a "*lone voice*" where few other people had an interest in this population.

"And the other point I just want to make is sometimes you feel like a lone voice out here kind of shouting about how important these things are. And I feel like I'm not on my own for a while." (PT7)

However, not all HCPs had this level of curiosity or interest about working with people with advanced dementia. Many of our participants provided teaching and education to others relating to dementia, but often with little positive engagement.

"I actually got onto the physio course for them for me to teach them and I did two consecutive years …..People weren't interested….. a room of 50 people just shut down, as if cognition and old and people with cognitive frailty was something they'd never come across." (PT1)

This was particularly reported amongst physiotherapists and there was a belief that occupational therapists were more involved in treating people with dementia or cognitive difficulties historically, although there was a feeling that this attitude was beginning to change. There was further evidence to support this with our participants discussing the role of physiotherapists in mental health teams being much less advanced than the role of occupational therapists.

"I was one of the first physiotherapist who was hired to work in a nursing home and in [country]…..I've worked a lot with nurses and caregivers and they've been really interested in what I could offer. I have [tried with] the physiotherapist. They weren't interested." (PT5)

There was considerable optimism amongst our participants that people with advanced dementia could effectively receive rehabilitation and other interventions, but this came from positive previous experience – thus their wisdom. It was notable that sharing this experience and positive outcomes was used as a way to engage other HCPs in treating people with advanced dementia.

Knowledge of negative outcomes for people with dementia also led HCPs to be more proactive in their approaches, often actively seeking out patients who would benefit from specialist interventions.

"….we've also changed our practice. Whenever anyone go to the to that trust, we've phoned and we make sure and we may actually go out." (PT10)

However, this optimism was often diminished by challenges relating to service pressures and limitation that the HCPs had to work within. Unsuitable ward environments were a particular challenge and the HCPs accepted the fact that the person with advanced dementia was unlikely to be able to improve in that setting and therefore focused their attention on ensuring they could be discharged to the most appropriate location to facilitate and rehabilitation. Where this optimism

For peer review only

was reduced, HCPs noted feeling very alone and isolated when trying to “fight” for people with dementia.

It was evident that our participants felt a significant amount of compassion towards their patients, particularly those with advanced dementia. There was discussion about the mutual upset that they felt when they didn’t feel people with dementia received optimal care and this was clearly upsetting to participants – some giving examples of poor care that they had experiened. There was a belief that people with advanced dementia were often stigmatised and labelled which prevented them from receiving the care that they needed and deserved.

“…there's this been this historic thing about ‘no rehab potential’. And the consistent negative labelling of our people that we work with…….’No capacity, no rehab potential behaviours that challenge aggressive.’ That that's always been the negative, negative and pejorative labelling.” (PT1)

There was also a feeling of guilt and frustration where a HCP was not able to provide the intervention they wanted to – usually due to resource pressures or the culture of the organisation. However, it was evident that the individuals attitude was key and their beliefs and engagement with people with advanced dementia had a pivotal role in determining what intervention was delivered.

“….attitude is huge. I think if you if you don't have the right attitude, nothing really follows….. three words that stand out for me are time, connection and value. So value in the person value in the carer value in their journey and where they've got to until now.” (PT7)

The interrelationship between the values of the HCP, the organisational culture and the knowledge of the HCP is detailed in Table 2. Our data suggests that in order for the person with dementia to have positive outcomes, all three variables need to be optimised.

Table 2 - detailing the interaction of factors and the outcomes for the person with dementia and the HCP

Values of the HCP	Organisational Culture	Knowledge of the HCP	Outcomes	
			For the person living with dementia	For the HCP
Good	Good	Good	Positive outcomes	Satisfaction and ability to make a positive improvement in care
Good	Good	Poor	Poor outcomes	Willingness and desire to improve outcomes, but insufficient knowledge to achieve good outcomes
Good	Poor	Poor	Poor outcomes	Frustration that the HCP values the person with dementia but has neither the positive culture within

				the organisation or the knowledge to make a positive change.
Good	Poor	Good	Poor outcomes	Frustration of HCP being unable to achieve good outcomes for the patient as the organisation does not support their interventions
Poor	Poor	Good	Poor outcomes	Unable to use the knowledge they have in an effective manner
Poor	Poor	Poor	Poor outcomes	Lack of interest or concern from HCP. Skills and knowledge deteriorate further.

Discussion

The aims of the study were to explore the rehabilitation principles when providing and delivering rehabilitation for people with advanced dementia. Our participants described the key aspects to consider the culture of rehabilitation and raised questions about what this means, but also what other people and HCPs perceive it to be. There was a common belief that knowledge was key and where there was a lack of knowledge about treating people with advanced dementia outcomes were poor – or people simply weren't offered interventions. However, there was a noted importance on the values of the HCP themselves and how these values could be directly influenced by the culture of the organisation and the knowledge about how best to manage people with advanced dementia.

Our data suggests an important relationship between these factors and for a person with advanced dementia to receive optimal interventions, there needs to be a symbiotic relationship between all three factors (Table 2). Where any one of the elements is missing, the person with dementia will not receive the care that they need.

Our participants reported that knowledge was a key domain to ensure people with advanced dementia receive optimal care, many giving examples of where a lack of knowledge resulted in poor outcomes for people with dementia. This particularly related to an understanding of dementia, but more specifically, how to ensure interventions were person centred. It was clear that lack of education relating to dementia and person-centred care approaches would significantly affect interventions. Lack of knowledge amongst healthcare professionals is inconsistent with one large study undertaken by Alzheimer's Disease International suggesting that 62% of healthcare professionals believed that dementia is a natural part of ageing and supported other studies findings stating the belief there was nothing that could be done to manage it [19, 20]. However, it is unclear

whether and how this relates to specific professions. Our data suggests a lack of undergraduate training for physiotherapists relating to dementia, however a Canadian study surveyed physiotherapists and found that levels of knowledge relating to dementia was high, but cited lack of confidence to manage behavioural and cognitive difficulties [21]. Interestingly, similar research exploring the attitudes of physiotherapy students found that 53% felt their academic training was sufficient to equip them with the skills to work with people with dementia [22], suggesting a reduction in confidence from undergraduate level to postgraduate level.

Our data suggests that the personal values, and thus the attitude, towards a person with advanced dementia was pivotal in determining the care that was delivered and received. Our participants were all experts in treating people with advanced dementia and therefore had compassion and interest in treating this population, however they reported numerous examples where poor personal values of the HCP negatively affected the person with dementia – and thus their outcomes. Research has explored the importance of providing education and training for healthcare professionals relating to dementia in the acute setting [23], with a highlighted requisite that this education supports the development of empathy and understanding relating to the person with dementia [23, 24]. However, much of this research focuses on attitudes in relation to knowledge rather than the person values of the HCP and how these influence the care provided.

The culture of the organisation was suggested to influence the HCPs values and conversely, HCPs with poor values relating to dementia could negatively affect the culture of the organisation. The culture of care has been linked to the value placed on the person with dementia [25], thus placing greater value on the person facilitates a culture which supports them. However, it has been suggested that education alone is not sufficient to facilitate this positive culture [23, 24]. Organisations need a strong positive leadership culture which promotes person centred care [26] and such leaders need to act as role models to demonstrate positive care. For people with complex needs, such as those with advanced dementia, situational leadership is recommended in the nursing literature [27] to support those with less experience. Lack of time and suitable environments were cited as being major barriers to facilitating effective interventions for people with advanced dementia and must be considered key to developing this positive culture.

Our data suggests that the inter-relationship between the knowledge and values of the HCP and the culture of the organisation are all key to successful outcomes. Our data (table 2) suggests that only positive attributes of all three domains will result in successful outcomes for people with dementia.

Conclusion

This qualitative study contributes to the knowledge about key domains to consider when providing rehabilitation interventions for people with advanced dementia. We propose that there are three key domains which must be optimised to ensure the best outcomes for people with advanced dementia. The organisational culture must support person centred care approaches, while providing sufficient resources to support interventions. The HCP must have appropriate dementia specific

For peer review only

knowledge and their personal values must align with the requirements for people with dementia. Should all key domains be optimised, the person with advanced dementia has potential to engage successfully with rehabilitation interventions. Further research is needed to determine the content of the actual interventions that are delivered.

Author Contributions

Conceptualization: AH.; methodology: AH, FM, VG; formal analysis: AH, FM, VG. ; data curation; AH, FM, VG..; writing—original draft: AH.; writing—review and editing: AH, FM, VG.; supervision: VG.; All authors have read and agreed to the published version of the manuscript.

Funding

This study is independent research funded by the National Institute for Health and Care Research (NIHR) School for Primary Care Research (119247) and supported by the NIHR Applied Research Collaboration South West Peninsula. The views expressed in this publication are those of the author(s) and not necessarily those of the National Institute for Health Research or the Department of Health and Social Care.

Institutional Review Board Statement

The study was conducted according to the guidelines of the Declaration of Helsinki, and approved by the and ethical approval was obtained from the University of Exeter's College of Medicine and Health Research Ethics Committee (reference 515745).

Informed Consent Statement

Informed consent was obtained from all subjects involved in the study.

Data Availability Statement

The datasets generated during and/or analyzed during the current study are available from the corresponding author upon reasonable request.

Acknowledgments

The authors thank all the participants who devoted their time to participate in this study.

For peer review only

Conflicts of Interest

The authors declare no conflict of interest.

References

1. Organization, W.H., *WHO global strategy on people-centred and integrated health services: interim report*. 2015, World Health Organization.
2. Organization, W.H., *Dementia: a public health priority*. 2012: World Health Organization.
3. Ahmadi-Abhari, S., et al., *Temporal trend in dementia incidence since 2002 and projections for prevalence in England and Wales to 2040: modelling study.* bmj, 2017. **358**.
4. Mitchell, S.L., *Advanced dementia.* New England Journal of Medicine, 2015. **372**(26): p. 2533-2540.
5. Banerjee, S., et al., *Quality of life in dementia: more than just cognition. An analysis of associations with quality of life in dementia.* Journal of Neurology, Neurosurgery & Psychiatry, 2006. **77**(2): p. 146-148.
6. Hall, A.J., S. Febrey, and V.A. Goodwin, *Physical interventions for people with more advanced dementia–a scoping review.* BMC geriatrics, 2021. **21**(1): p. 1-10.
7. Forbes, D., et al., *Physical activity programs for persons with dementia.* Cochrane Database of Systematic Reviews, 2008(3).
8. Lamb, S., et al., *Aerobic and strength training exercise programme for cognitive impairment in people with mild to moderate dementia: the DAPA RCT.* Health Technology Assessment, 2018. **22**(28).
9. Blondell, S.J., R. Hammersley-Mather, and J.L. Veerman, *Does physical activity prevent cognitive decline and dementia?: A systematic review and meta-analysis of longitudinal studies.* BMC public health, 2014. **14**(1): p. 1-12.
10. Groot, C., et al., *The effect of physical activity on cognitive function in patients with dementia: a meta-analysis of randomized control trials.* Ageing research reviews, 2016. **25**: p. 13-23.
11. Engel, G.L., *The need for a new medical model: a challenge for biomedicine.* Science, 1977. **196**(4286): p. 129-136.
12. Scerri, A., A. Innes, and C. Scerri, *Healthcare professionals' perceived challenges and solutions when providing rehabilitation to persons living with dementia—A scoping review.* Journal of Clinical Nursing. **n/a**(n/a).
13. Mitchell, R., et al., *Access to rehabilitation services for older adults living with dementia or in a residential aged care facility following a hip fracture: healthcare professionals' views.* Disability and Rehabilitation, 2021. **43**(6): p. 834-845.

For peer review only

14. McCarroll, C., C. van't Riet, and M. Halter, *How is the emerging role of domiciliary physiotherapists who treat residents with dementia in nursing homes perceived by allied health professionals? A phenomenological interview study.* Health & Social Care in the Community, 2020. **28**(1): p. 279-290.
15. Borbasi, S., et al., *Health professionals' perspectives of providing care to people with dementia in the acute setting: Toward better practice.* Geriatric Nursing, 2006. **27**(5): p. 300-308.
16. Røsvik, J. and A.M.M. Rokstad, *What are the needs of people with dementia in acute hospital settings, and what interventions are made to meet these needs? A systematic integrative review of the literature.* BMC Health Services Research, 2020. **20**(1): p. 723.
17. Hall, A.J., et al., *Community physiotherapy for people with dementia following hip fracture: Fact or fiction? A qualitative study.* Dementia, 2020. **19**(8): p. 2750-2760.
18. Clarke, V. and V. Braun, *Thematic analysis*, in *Encyclopedia of critical psychology*. 2014, Springer. p. 1947-1952.
19. Lynch, C., *World Alzheimer Report 2019: Attitudes to dementia, a global survey: Public health: Engaging people in ADRD research.* Alzheimer's & Dementia, 2020. **16**: p. e038255.
20. Zhao, W., et al., *Hospital healthcare professionals' knowledge of dementia and attitudes towards dementia care: A cross‐sectional study.* Journal of Clinical Nursing, 2022. **31**(13-14): p. 1786-1799.
21. Hunter, S.W. and A. Divine, *Understanding the factors influencing physiotherapists' attitudes towards working with people living with dementia.* Physiotherapy Theory and Practice, 2021. **37**(12): p. 1448-1455.
22. Hunter, S.W., et al., *Physiotherapy students' attitudes toward working with people with dementia: A cross-sectional survey.* Physical & Occupational Therapy In Geriatrics, 2020. **38**(1): p. 56-66.
23. Cowdell, F., *Care of older people with dementia in an acute hospital setting.* Nursing Standard (through 2013), 2010. **24**(23): p. 42.
24. Moyle, W., et al., *Care staff attitudes and experiences of working with older people with dementia.* Australasian Journal on Ageing, 2011. **30**(4): p. 186-190.
25. Houghton, C., et al., *Healthcare staffs' experiences and perceptions of caring for people with dementia in the acute setting: Qualitative evidence synthesis.* International Journal of Nursing Studies, 2016. **61**: p. 104-116.
26. Rokstad, A.M.M., et al., *The role of leadership in the implementation of person-centred care using Dementia Care Mapping: a study in three nursing homes.* Journal of Nursing Management, 2015. **23**(1): p. 15-26.
27. LYNCH, B.M., B. McCORMACK, and T. McCANCE, *Development of a model of situational leadership in residential care for older people.* Journal of Nursing Management, 2011. **19**(8): p. 1058-1069.

For peer review only

COREQ (COnsolidated criteria for REporting Qualitative research) Checklist

A checklist of items that should be included in reports of qualitative research. You must report the page number in your manuscript where you consider each of the items listed in this checklist. If you have not included this information, either revise your manuscript accordingly before submitting or note N/A.

Topic	Item No.	Guide Questions/Description	Reported on Page No.
**Domain 1: Research team and reflexivity**			
Personal characteristics			
Interviewer/facilitator	1	Which author/s conducted the interview or focus group?	
Credentials	2	What were the researcher's credentials? E.g. PhD, MD	
Occupation	3	What was their occupation at the time of the study?	
Gender	4	Was the researcher male or female?	
Experience and training	5	What experience or training did the researcher have?	
Relationship with participants			
Relationship established	6	Was a relationship established prior to study commencement?	
Participant knowledge of the interviewer	7	What did the participants know about the researcher? e.g. personal goals, reasons for doing the research	
Interviewer characteristics	8	What characteristics were reported about the inter viewer/facilitator? e.g. Bias, assumptions, reasons and interests in the research topic	
**Domain 2: Study design**			
Theoretical framework			
Methodological orientation and Theory	9	What methodological orientation was stated to underpin the study? e.g. grounded theory, discourse analysis, ethnography, phenomenology, content analysis	
Participant selection			
Sampling	10	How were participants selected? e.g. purposive, convenience, consecutive, snowball	
Method of approach	11	How were participants approached? e.g. face-to-face, telephone, mail, email	
Sample size	12	How many participants were in the study?	
Non-participation	13	How many people refused to participate or dropped out? Reasons?	
Setting			
Setting of data collection	14	Where was the data collected? e.g. home, clinic, workplace	
Presence of non-participants	15	Was anyone else present besides the participants and researchers?	
Description of sample	16	What are the important characteristics of the sample? e.g. demographic data, date	
Data collection			
Interview guide	17	Were questions, prompts, guides provided by the authors? Was it pilot tested?	
Repeat interviews	18	Were repeat inter views carried out? If yes, how many?	
Audio/visual recording	19	Did the research use audio or visual recording to collect the data?	
Field notes	20	Were field notes made during and/or after the inter view or focus group?	
Duration	21	What was the duration of the inter views or focus group?	
Data saturation	22	Was data saturation discussed?	
Transcripts returned	23	Were transcripts returned to participants for comment and/or	

For peer review only

Topic	Item No.	Guide Questions/Description	Reported on Page No.
		correction?	
**Domain 3: analysis and findings**			
Data analysis			
Number of data coders	24	How many data coders coded the data?	
Description of the coding tree	25	Did authors provide a description of the coding tree?	
Derivation of themes	26	Were themes identified in advance or derived from the data?	
Software	27	What software, if applicable, was used to manage the data?	
Participant checking	28	Did participants provide feedback on the findings?	
Reporting			
Quotations presented	29	Were participant quotations presented to illustrate the themes/findings? Was each quotation identified? e.g. participant number	
Data and findings consistent	30	Was there consistency between the data presented and the findings?	
Clarity of major themes	31	Were major themes clearly presented in the findings?	
Clarity of minor themes	32	Is there a description of diverse cases or discussion of minor themes?	

Developed from: Tong A, Sainsbury P, Craig J. Consolidated criteria for reporting qualitative research (COREQ): a 32-item checklist for interviews and focus groups. *International Journal for Quality in Health Care*. 2007. Volume 19, Number 6: pp. 349 – 357

Once you have completed this checklist, please save a copy and upload it as part of your submission. DO NOT include this checklist as part of the main manuscript document. It must be uploaded as a separate file.

For peer review only

TOPIC GUIDE

Researcher to introduce self and explain process of focus group, group rules, confidentiality, permission to record, right to withdraw.

**Topic**	**Prompts**
**Introduction – each member**<br><br>Please can you tell me a bit about your experience of working with people with advanced dementia and any challenges that you experience supporting them	Profession<br><br>Interest in PwD<br><br>System/organisational pressures<br><br>Individual patient difficulties<br><br>Carer
**Treatments**<br><br>What things are important to ensure that a treatment with a person with advanced dementia is effective?	Who present?<br><br>Involvement of family/carers<br><br>Duration<br><br>Type of intervention<br><br>Patient outcomes / QoL<br><br>Daily routines
**Location**<br><br>What are the key considerations when providing healthcare interventions in a nursing home compared to at home?	Environment<br><br>Formal carers – engagement, education, knowledge<br><br>Equipment
**Communication**<br><br>What factors are important to consider when communicating with a person with advanced dementia?	Body language<br><br>Tone

For peer review only

	Language
**Training/education**<br><br>Therapists with a variety of experience will be treating people with advanced dementia, therefore what education or skills are needed to be able to treat people with advanced dementia?	Undergraduate learning<br><br>Formal learning<br><br>Experiential learning

Is there something else that I have not asked you about, that you would like to tell me about?

Thank you

For peer review only

BMJ Open

A qualitative study exploring health care professionals' perceptions of providing rehabilitation for people with advanced dementia

Journal:	*BMJ Open*
Manuscript ID	bmjopen-2023-072432.R2
Article Type:	Original research
Date Submitted by the Author:	30-Jun-2023
Complete List of Authors:	Hall, Abigail J.; University of Exeter, Public Health and Sports Science<br>Manning, Fay; University of Exeter Medical School, Department of Medical Imaging<br>Goodwin, Victoria; University of Exeter, Public Health and Sports Science
**Primary Subject Heading**:	Rehabilitation medicine
Secondary Subject Heading:	Qualitative research
Keywords:	Dementia, REHABILITATION MEDICINE, QUALITATIVE RESEARCH

SCHOLARONE™ Manuscripts

I, the Submitting Author has the right to grant and does grant on behalf of all authors of the Work (as defined in the below author licence), an exclusive licence and/or a non-exclusive licence for contributions from authors who are: i) UK Crown employees; ii) where BMJ has agreed a CC-BY licence shall apply, and/or iii) in accordance with the terms applicable for US Federal Government officers or employees acting as part of their official duties; on a worldwide, perpetual, irrevocable, royalty-free basis to BMJ Publishing Group Ltd ("BMJ") its licensees and where the relevant Journal is co-owned by BMJ to the co-owners of the Journal, to publish the Work in this journal and any other BMJ products and to exploit all rights, as set out in our licence.

The Submitting Author accepts and understands that any supply made under these terms is made by BMJ to the Submitting Author unless you are acting as an employee on behalf of your employer or a postgraduate student of an affiliated institution which is paying any applicable article publishing charge ("APC") for Open Access articles. Where the Submitting Author wishes to make the Work available on an Open Access basis (and intends to pay the relevant APC), the terms of reuse of such Open Access shall be governed by a Creative Commons licence – details of these licences and which Creative Commons licence will apply to this Work are set out in our licence referred to above.

Other than as permitted in any relevant BMJ Author's Self Archiving Policies, I confirm this Work has not been accepted for publication elsewhere, is not being considered for publication elsewhere and does not duplicate material already published. I confirm all authors consent to publication of this Work and authorise the granting of this licence.

For peer review only

A qualitative study exploring health care professionals' perceptions of providing rehabilitation for people with advanced dementia

Abigail J Hall (corresponding author) BSc, MSc, PhD, MCSP

Public Health and Sports Science, University of Exeter, Heavitree Road, Exeter, EX1 2LU

a.hall4@exeter.ac.uk

Fay Manning BSc, MSc, PhD

Department of Medical Imaging, University of Exeter, Heavitree Road, Exeter, EX1 2LU

Victoria A Goodwin, MBE, BSc, MSc, PhD, FCSP,

Public Health and Sports Science, University of Exeter, Heavitree Road, Exeter, EX1 2LU

For peer review only

Abstract

Objectives

The aim of this study was to explore health care professionals' principles for providing and delivering rehabilitation interventions for people with advanced dementia.

Design

This was a qualitative study with three focus groups undertaken virtually. The data was analysed using a process of reflexive thematic analysis in order to gain an in depth understanding of rehabilitation principles for this population.

Setting and Participants

20 healthcare professionals who were specialists in treating and rehabilitating people with advanced dementia were recruited. These healthcare professionals had a wide range of experience in a variety of different settings including primary care, secondary care as well as specialist mental health teams. Purposive sampling focused on the requirement for participants to have significant experience of treating people with dementia. Participants were from the UK and Denmark. Data collection was undertaken during August and September 2022.

Results

Three overarching themes were developed following analysis – organisational culture, knowledge and personal values of the health care professional. The first explored how the culture of an organisation affects a person with advanced dementia as well as the healthcare professional. The organisation needed to promote positive approaches to person centred care and provide effective situational leadership to embed such approaches. Knowledge was a key consideration and was closely linked to the personal values of the healthcare professional, which formed the final theme. This study suggests that the interrelationship of these three factors influences the outcomes for the person with dementia and effective outcomes required consideration of all domains.

Conclusions

Effective interventions for people with advanced dementia require the healthcare professional to have the knowledge about dementia and positive personal values, but the culture of the

For peer review only

organisation is also key to ensure that the healthcare professional is able to deliver successful interventions.

Article Summary

Strengths and limitations of this study

- This work was informed and supported by an active Patient Participation Involvement group – including collaborating with designing the study and discussing the findings.
- The methods of data collection and analysis were robust and followed clear guidance for undertaking such qualitative work.
- The study was reported using COREQ (see supplementary material) to ensure clear and transparent methods and analysis.
- Although this was a small-scale qualitative study with a limited range of healthcare professionals taking part- which could impact on generalisability- sampling was deliberately specific and engaged HCPs who were specialists in treating people with very advanced dementia. This reduced the potential sample size, as there is a limited sample of people due to the specialist nature of interventions.

Introduction

Dementia is a neurological disorder resulting in physical and cognitive decline, found typically in older adults (1, 2). It is estimated that by 2040, there will be 1.2 million people with dementia living in the UK – a 57% increase from 2016 (3), suggesting that dementia will become a national – and global – challenge to healthcare services. Advanced dementia is characterised by profound communication, memory and physical disturbances (4), which often result in significant impairments in a person's ability to undertake activities of daily living and a perceived decline in quality of life (5).

A variety of healthcare professionals (HCPs) can be involved in trying to reduce the physical declines associated with advanced dementia, including physiotherapists and occupational therapists. However, there remains little evidence to support physical interventions for people with dementia (6-10). This lack of evidence is particularly evident for people with more severe dementia, where physical interventions to improve physical ability – or reduce the speed of decline – are largely not understood (6).

There is much debate about the approach that is suitable for people with advanced dementia, with greater emphasis being placed on adopting a biopsychosocial model, where functioning and participation is key. Some 60 years ago, a key paper - "The need for a new model: A challenge for biomedicine" (11) argued that the dominant biomedical model of disease was insufficient for a complete understanding of health. It was stated that a biomedical approach ''*assumes disease to be fully accounted for by deviations from the norm of measurable, biological variables. It leaves no room within its framework for the social, psychological and behavioural dimensions of illness''* (p. 130). Considering not only biological components, but also the individual and societal contexts of the individual's experiences is vital in the rehabilitation of people living with dementia to ensure a person-centred approach to achieve what is meaningful to the individual.

However, the challenge that this poses to healthcare professionals is poorly understood – especially for people living with more advanced dementia (6, 12-14).

The experience of health care professionals treating people with dementia has been explored in a variety of settings (15, 16), including residential aged care facilities (16, 17) and acute settings (18). A recent review exploring the experiences of caring for people with dementia in acute settings has highlighted the importance of developing knowledge of person centred approaches to care, however research has also highlighted a lack of understanding about the importance of the environment and how this may impact care (19). However, evidence suggests that providing rehabilitation interventions for people with more advanced dementia is different to those with more mild forms (6, 14). Thus, the aims of the study were to explore the rehabilitation principles of HCPs providing and delivering rehabilitation interventions for people with advanced dementia in a variety of settings including acute, community and residential care.

Methods

Design

During August and September 2022, three semi-structured focus groups were undertaken with a range of HCPs. These health professionals were all specialists in the management or rehabilitation of people living with advanced dementia. The focus groups were undertaken virtually to reduce barriers due to geographical limitations and enabled international specialists to contribute. The study has been reported according to the COREQ guidelines (see supplementary material).

Setting and participants

A purposive sampling strategy was employed to seek HCPs who specialise in working with people with advanced dementia. A targeted social media advert sought professionals with advanced skills and pre-existing networks were drawn upon to recruit participants. Some of these participants were known to the researchers in a professional capacity due to previous work undertaken in this area. This did not affect the data collection.

Inclusion criteria:

- Specialist working with people with dementia as a physiotherapist, occupational therapist, psychologist, nurse, or other related profession. ("Specialist" refers to the level of experience and training that an HCP has and an expectation that they are working in an advanced role relating to the care of people with dementia)

Participants who responded to the social media advert or initial email were sent a follow up email with the participant information sheet attached. At this point they were offered an opportunity to discuss the study further if they had any particular questions. If they were happy to take part in the study, they were asked to provide informed consent prior to the commencement of the focus group. Participants were informed that they were under no obligation to participate in the focus group and

For peer review only

they may withdraw from it at any time, without any negative consequence, up until the point where the data were fully anonymised.

Provided the participant met the inclusion criteria, all participants who consented to take part were invited to join a focus group. Recruitment continued until sufficient data was obtained to be able to answer the aim of the study and no new data was being generated.

Data collection

The focus groups aimed to explore the challenges and techniques required to effectively manage people with more advanced dementia, while also considering the principles of rehabilitation for this population. The focus groups were facilitated by two experienced qualitative researchers (AH and FM). FM acted as a 'second facilitator/observer', taking notes and making observations pertaining to interaction of the group. Participants were all encouraged to give their opinions and thoughts during the meeting. To ensure that everybody was given the opportunity to contribute, each participant was asked whether they had further detail to add to any of the topics before moving on to the next. Both researchers have undertaken training courses pertaining to qualitative analysis and have published multiple qualitative papers.

Microsoft Teams was used to undertake the focus groups and they were recorded and automatically transcribed. The transcription function has been used by the researcher and found to be accurate, although the transcripts were all checked for accuracy. The focus groups lasted for a maximum of 90 minutes and included time for participants to introduce themselves and their roles to other members of the group. The participants were informed that this study formed part of a larger piece of work aiming to develop interventions for people with advanced dementia. A semi-structured guide (see supplementary material) was used to guide the conversation of the focus groups. The topic guide was not piloted prior to the initial focus group as it was anticipated that it could be adapted for subsequent focus groups if needed. This was not required and the same topic guide was used for all interviews. The data was anonymised so that individual participants could not be identified, and all participants consented to their data being transcribed and analysed. Transcripts were not reviewed by participants. The data collection from the focus groups provided rich and detailed data and no further follow-up interviews were deemed necessary although people were offered the opportunity to discuss any further points following completion of the focus group.

For peer review only

Patient and Public Involvement (PPI)

A patient and public involvement (PPI) group was developed to help inform the research undertaken during this study and as part of a wider programme of work. The group has been evolving and developing and now consists of people with a variety of experiences of dementia. The group includes people living with dementia, carers of people with dementia and two health care professional who have personal experience of dementia.

Initial discussions focused on the challenges they had experienced with getting rehabilitation for people who have dementia – especially when the person had advanced dementia or was living in a care home. Several themes generated from the PPI members formed part of the focus group

discussion. Due to practical challenges, none of the PPI group were able to participate in the focus groups, but discussion was held with representatives following the groups and helped guide the analysis and results.

Analysis

Analysis commenced after the first focus group was completed and continued throughout the following focus groups. As the data collection and initial analysis ran simultaneously, arising themes or gaps could be probed accordingly in the remaining focus groups.

NVivo 11 (QSR International) was used to organize and code the data. This facilitated a process of reflexive thematic analysis to be undertaken - as suggested by Braun and Clarke (20). Familiarisation of the data involved reading and re-reading the transcripts. A process of systematic open coding was then commenced and initial ideas for themes were noted. Following this open coding, initial themes were generated, discussed and explored with the wider research team. These discussions led to further refinement of the themes, and definitions written to ensure clarity between the research team. For example, there were multiple codes relating to participants using positive success stories to educate others, this was collated into a minor theme of "optimism/wisdom" which was then classified within the "personal values of the HCP". This process was repeated for all the data until themes were developed. This process was an iterative process and resulted in several refinements of named themes. These themes were then discussed with the patient and public involvement group who were supporting and collaborating on the overarching project.

Ethics

Ethical approval was obtained from the University of Exeter's College of Medicine and Health Research Ethics Committee (reference 515745).

Findings

20 healthcare professionals took part in three focus groups (Table 1). Only one person who had initially expressed an interest in taking part did not reply to the meeting invite and therefore did not take part. Following the third focus group, it was felt that no new themes were occurring and therefore it was believed that theoretical data saturation had been reached and no further focus groups were planned. There were no withdrawals from the study at any stages. The HCPs reported an average of eighteen years of experience and an average of thirteen years specializing specifically with people with dementia. The settings of their experience were broad, including in-patient settings, out-patient clinics, patients' own homes and residential/nursing settings.

Table 1 - characteristics of participants (n=20)

**Sex**	**Female**	**19**
	Male	1

For peer review only

**Profession**	Physiotherapist	14
	Occupational Therapist	3
	Nurse	2
	Psychologist	1
**NHS Grade / Banding**	6	6
	7	8
	8+	2
	N/A	4
**Years of clinical experience**	0-5	1
	6 - 10	2
	11-15	2
	16 - 20	3
	21 - 25	6
	26 - 30	2
	30+	3
	Not reported	1
**Years of experience working with people with advanced dementia**		
	0-5	4
	6 - 10	2
	11-15	6
	16 - 20	5
	21 - 25	2
	26 - 30	1
	30+	0
**Location**	England	13
	Wales	2
	Scotland	4
	Denmark	1
**% of caseload of people with dementia**		
	0 - 25	1
	26 - 50	1
	51 - 75	6
	76 - 100	8
	Not currently working clinically	4
**Locations of work**	In-patient	12
	Out-patient	4
	Care home/residential homes	11
	Own homes	11
	Other	2

The principles of rehabilitation were explored for people with advanced dementia and three overarching themes were developed following analysis. These were 'organisational culture', 'knowledge about dementia' and 'personal values of the HCPs'. The first theme explores how

For peer review only - http://bmjopen.bmj.com/site/about/guidelines.xhtml

organisational culture may affect the person with advanced dementia as well as the HCP working within a system or organisation. Secondly, knowledge of dementia was considered to be a key consideration when exploring the rehabilitation of people with advanced dementia, but this was closely linked to the personal values of the healthcare professional, which formed the final theme. Each theme will be explored in further detail and then the interaction between them considered.

Organisational Culture

Our participants discussed how there was a varied understanding of what "rehabilitation" means to people and how this differing understanding led to people with advanced dementia receiving a wide variety of interventions. This often related to understanding what the purpose of rehabilitation was, from seeking to gain functional improvements to preventing decline. There was also discussion about the consideration of how services and organisations' cultures affected the interventions that were delivered.

Determining what the term "rehabilitation" means was reported to be challenging for healthcare professionals generally. What an organisation considered to be rehabilitation would significantly affect what was delivered. Our participants were very clear that for people with advanced dementia, tasks that were perceived to be 'simple' were actually very important and, although not deemed as "traditional rehabilitation" were actually important aspects of rehabilitation.

"we talk a lot in our training about the concept of rehab as what we find is if…. they're not looking to get them back up on their feet, then that's not rehab. Whereas my argument that is actually for somebody with quite an advanced dementia, we have help them into a sitting position, can have massive functional benefits for them. So that in itself is rehab" (PT9)

The term "traditional rehabilitation" was discussed in all focus groups and led back to the underlying biomedical principles that historically have been adopted by many healthcare professionals. This approach was focused on the injury or illness.

"…..[we] know all about anatomy and physiology and this is how to treat a broken leg. This is how to treat a back pain. But they don't actually get taught how to embrace that full person centered approach to things." (PT7)

There was also a noted discomfort that HCPs felt that they had to justify doing things differently – and thus outcome measures were described as being vital to "prove effectiveness" of their interventions. Often organisations demanded these outcome measures to prove their services were effective. It was also discussed that often it was only the physical interventions that were perceived to be treatment – to such an extent that only the physical interventions were often documented. Our participants were very clear that these "non-traditional" approaches needed to be clearly documented to justify their interventions.

"I think is well, it's about how we document that because we don't always document those times when we've gone in and not been able to engage with somebody. We maybe say they weren't able to engage in our documentation, but

we don't say we spent time engaging with that person and it resulted in an improved rapport, improved connection." (PT7)

Complexity of patients was noted as requiring a different approach and people with advanced dementia were recognised as being highly complex and therefore needing care to be adapted accordingly.

"We're trying to pretend that we haven't got a complex system….which a complex patient is.... we're still trying to imagine that we've got a simple machine where if you put one thing in change, one of the dials, you're gonna get a completely different product out. And so it goes right back to undergraduate education and absolutely rethinking our model of physiotherapy." (PT1)

It was discussed that the focus of rehabilitation has often been to make a tangible improvement after an illness or injury which initially resulted in a decline in physical function. However, our participants questioned this and reported that often for people with advanced dementia, rehabilitation could be considered a slowing of the inevitable physical decline that is associated with the condition.

"….because it's a degenerative disorder and …. the processes that these patients will deteriorate…..it's not just about improving things for them, but maintaining what they're able to do for as long as physically possible as well" (PT6)

While there was a focus on preventing decline, there was also belief that people with advanced dementia can improve physically – therefore this should be a focus of the intervention. However, it was clear that this potential to improve physically required timely – and appropriately targeted – interventions, often before there was significant physical decline.

The unclear nature of interventions for people with advanced dementia makes it difficult to determine their trajectory of physical recovery, however, our HCPs felt very strongly that people needed to be given an opportunity to receive rehabilitation. In the acute setting, giving these opportunities was challenging due to the focus on discharging patients to ensure good patient "flow". Despite this challenge, our participants were determined to offer as much as they possibly could, but they recognised this was often insufficient.

"Yeah, time, time for us… how long you can spend with the patient. Obviously you know you have so many patients to split your time between. But our biggest time pressure is discharge pressures." (PT13)

The influence of the service or organisation that a person was working within had a significant impact on the interventions that could be delivered. The way that an organisation did, or in some cases did not, adapt their environments and policies to suit a person with advanced dementia would influence their journeys.

"The acute sector is massively, massively limited in what it can provide people with dementia…. the environment is completely wrong. It's wrong for people without dementia. It's completely wrong for people with dementia, so of course they're not gonna give their best selves." (PT4)

For peer review only

While the acute setting had a significant focus on patient flow, the actual environment was deemed very unsuitable for people with advanced dementia. Noise, inappropriate lighting and distractions would often add to challenging behaviours that the person might display. Organisations needed to ensure their environments were suitable.

"we do seem to have a bit of an issue, I call it beige bays, we create these spaces and we set people in them and we say 'now you stay there for six hours, 10 hours, 12 hours and only get up if you need to go to the toilet'. We….actively deprive people of activity during their stay and then get really shocked why they start getting really curious/bored out of their brains and wanting to get up and moving." (OT2)

Organisations often were risk averse and fear of people falling and injuring themselves led to risk averse behaviours such as discouraging mobility or keeping people in bed, but HCPs feared this and actively tried to overcome these challenges. Discouraging activity led to deconditioning and deterioration of function which was considered a significant risk of harm in itself.

"I don't like the term deconditioning, because I think it's completely poor care and lack of facilitating to function… We've allowed this this appalling situation to happen where when our older people go into hospital, they deteriorate almost in front of in fact definitely in front of our eyes." (PT1)

There was a frustration that often people with advanced dementia were restricted from undertaking "risky" activities, but there was little emphasis placed on how this would affect the person from an emotional perspective.

"I hear people talking about the talking about the risk of falling over the risk of pressure ulcers, that risk of infection. We have huge risks to personhood, to selfhood, to self esteem, to independence, to the ability to live independently." (PT1)

Our HCPs also reported frustration with arbitrary timescales that patients were given to receive an intervention. Whether this was due to waiting lists for community services which were reported to be up to 40 weeks in some services, to the time limited services such as in-patient intermediate care or rehabilitation.

"….if you're gonna give people time, you need to look at the whole system, that of rehab….who came up with six weeks? I've got no idea. But that seems to be "if you can't get it and six weeks, then there's no point really" attitude." (PT9)

Knowledge relating to dementia

Knowledge relating to dementia and a good understanding of person centred care was deemed vital to be able to effectively treat a person with advanced dementia. Where there was a lack of knowledge, there were often poor outcomes for the person.

For peer review only

Knowledge relating to dementia was seen as being key to providing effective interventions. Our HCPs described challenges in gaining this knowledge, with many relying on experience and learning “on the job”. Undergraduate education for HCPs was universally felt to be inadequate and therefore, postgraduate education was vital, although difficult to access.

> *“The standard of university education on dementia for physios in particular and frailty is really, really poor and there's a lot and a lot of education that us more experienced physios need to be doing…. and that's why the words “no rehab potential” ….[are] still out there. Because there's not a lot of education around frailty and older people” (PT4)*

HCPs working in mental health settings found access to specific mental health training more accessible generally, although this was often generic rather than profession specific – this was particularly reported by physiotherapists who reported a lack of physiotherapy specific dementia training. There was a universal feeling amongst our participants that a large element of their role was about teaching and educating others – about dementia itself, but also the importance of delivering person centred care approaches to interventions. This related to other HCPs, but also to patients themselves and their carers.

A lack of knowledge and understanding was deemed to precipitate the belief that people with dementia had “no rehabilitation potential”. Indeed, this term was reported to be used very commonly.

> *“the staff who are labelling individuals of “no rehab potential” are often the less experienced staff ….. making a snapshot sort of decision within 10 minutes or five minutes.” (PT2)*

Person centred approaches to care were discussed in relation to a person with advanced dementia, but also in terms of how treatments should be adapted and personalised for everybody.

> *“It doesn't really matter if a patient has advanced dementia or if they're me, a 28 year old that's had knee surgery. If an exercise makes no sense to me, or if it's boring, or if it's not gonna help me get back to what I want to do, I'm probably not gonna do it. And really, if we make healthcare better for those with advanced dementia, we're gonna make it better for everyone, because what we're talking about there is personalized care, isn't it?” (PT4)*

This emphasis on ensuring that interventions were tailored to individuals was reported to be even more important to a person with advanced dementia, but understanding how to tailor interventions was variable and influenced by external factors such as time and availability of resources.

> *“…that time pressure because I think patients feel it and I think I might, you know, [allied health profession] colleagues feel it as well because literally as soon as people are either hitting a rehab ward or getting into an [intermediate care] bed, you're looking at discharge planning before you've even really got to the crux of that rehab” (PT9)*

Our HCPs reported that people were very aware of the concepts of person-centred approaches to care, but these were often seen as gold standard and their services were not always deemed able to

For peer review only

achieve this. Nor did they always have a good understanding about how to actually deliver such approaches. There was also suggestion that person centred care was not taught at undergraduate level in some professional groups, and this was something that had to be learnt as a post-graduate.

Personal values of the HCP

It was evident that the personal values of HCPs providing care for people with advanced dementia could significantly impact on the delivery of that care.

An interest in, and taking time, to get to know a person was deemed vital to be able to provide interventions to people with advanced dementia. However, a natural curiosity was evident amongst our participants. Where little formal education had been received, their curiosity to discover "what works" led them to a particular interest and passion for treating people with dementia. However, this was deemed to be the exception rather than the rule and many of our participants reported feeling like a "*lone voice*" where few other people had an interest in this population.

"And the other point I just want to make is sometimes you feel like a lone voice out here kind of shouting about how important these things are. And I feel like I'm not on my own for a while." (PT7)

However, not all HCPs had this level of curiosity or interest about working with people with advanced dementia. Many of our participants provided teaching and education to others relating to dementia, but often with little positive engagement.

"I actually got onto the physio course for them for me to teach them and I did two consecutive years …..People weren't interested….. a room of 50 people just shut down, as if cognition and old and people with cognitive frailty was something they'd never come across." (PT1)

This was particularly reported amongst physiotherapists and there was a belief that occupational therapists were more involved in treating people with dementia or cognitive difficulties historically, although there was a feeling that this attitude was beginning to change. There was further evidence to support this with our participants discussing the role of physiotherapists in mental health teams being much less advanced than the role of occupational therapists.

"I was one of the first physiotherapist who was hired to work in a nursing home and in [country]…..I've worked a lot with nurses and caregivers and they've been really interested in what I could offer. I have [tried with] the physiotherapist. They weren't interested." (PT5)

There was considerable optimism amongst our participants that people with advanced dementia could effectively receive rehabilitation and other interventions, but this came from positive previous experience – their wisdom. It was notable that sharing this experience and positive outcomes was used as a way to engage other HCPs in treating people with advanced dementia.

Knowledge of negative outcomes for people with dementia also led dementia specialist HCPs to be more proactive in their approaches, often actively seeking out patients who would benefit from specialist interventions.

For peer review only

"….we've also changed our practice. Whenever anyone go to the to that trust, we've phoned and we make sure and we may actually go out." (PT10)

However, this optimism was often diminished by challenges relating to service pressures and limitation that the HCPs had to work within. Unsuitable ward environments were a particular challenge and the HCPs accepted the fact that the person with advanced dementia was unlikely to be able to improve in that setting and therefore focused their attention on ensuring they could be discharged to the most appropriate location to facilitate rehabilitation. Where this optimism was reduced, HCPs noted feeling very alone and isolated when trying to "fight" for people with dementia.

It was evident that our participants felt a significant amount of compassion towards their patients. There was discussion about the mutual upset that they felt when people with dementia received sub-optimal care, and this was clearly upsetting to participants – some giving examples of poor care that they had experienced. There was a belief that people with advanced dementia were often stigmatised and labelled, which prevented them from receiving the care that they needed and deserved.

"…there's this been this historic thing about 'no rehab potential'. And the consistent negative labelling of our people that we work with…….'No capacity, no rehab potential behaviours that challenge aggressive.' That that's always been the negative, negative and pejorative labelling." (PT1)

There was also a feeling of guilt and frustration where an HCP was not able to provide the intervention they wanted to – usually due to resource pressures or the culture of the organisation. However, it was evident that the individuals' attitude was key and their beliefs and engagement with people with advanced dementia had a pivotal role in determining what intervention was delivered.

"….attitude is huge. I think if you if you don't have the right attitude, nothing really follows….. three words that stand out for me are time, connection and value. So value in the person value, in the carer value, in their journey and where they've got to until now." (PT7)

The interrelationship between the values of the HCP, the organisational culture and the knowledge of the HCP is detailed in Table 2. Our data suggests that in order for the person with dementia to have positive outcomes, all three variables need to be optimised.

Table 2 -theoretical model detailing the interaction of factors and the outcomes for the person with dementia and the HCP

Values of the HCP	Organisational Culture	Knowledge of the HCP	Outcomes	
			For the person living with dementia	For the HCP
Good	Good	Good	Positive outcomes	Satisfaction and ability to make a positive improvement in care

For peer review only

Good	Good	Poor	Poor outcomes	Willingness and desire to improve outcomes, but insufficient knowledge to achieve good outcomes
Good	Poor	Poor	Poor outcomes	Frustration that the HCP values the person with dementia but has neither the positive culture within the organisation or the knowledge to make a positive change.
Good	Poor	Good	Poor outcomes	Frustration of HCP being unable to achieve good outcomes for the patient as the organisation does not support their interventions
Poor	Poor	Good	Poor outcomes	Unable to use the knowledge they have in an effective manner
Poor	Poor	Poor	Poor outcomes	Lack of interest or concern from HCP. Skills and knowledge deteriorate further.

Discussion

The aim of the study was to explore the rehabilitation principles when providing and delivering rehabilitation for people with advanced dementia. Our participants described the key aspects to consider the culture of rehabilitation and raised questions about what this means, but also what other people and HCPs perceive it to be.

Our data suggests an important relationship between these factors and for a person with advanced dementia to receive optimal interventions there needs to be a symbiotic relationship between all three factors (Table 2). Where any one of the elements is missing, the person with dementia will not receive the care that they need. However, there was a noted importance on the values of the HCP themselves and how these values could be directly influenced by the culture of the organisation and the knowledge about how best to manage people with advanced dementia.

There was a common belief that knowledge about treating people with advanced dementia was key and where there was a lack of knowledge outcomes were poor – or people simply weren't offered interventions. This knowledge related both to the pathogenesis of dementia, how to provide care for

the person, but also how interventions could be suitably tailored to a accommodate the persons' cognitive abilities.

Knowledge about dementia amongst healthcare professionals is inconsistent, with one large study undertaken by Alzheimer's Disease International suggesting that 62% of healthcare professionals believed that dementia is a natural part of ageing and supported other studies findings that there was nothing that could be done to manage it (21, 22). However, it is unclear whether, and how, this relates to specific professions. Our data suggests a lack of undergraduate training for physiotherapists relating to dementia. A Canadian study surveyed physiotherapists and found that levels of knowledge relating to dementia was high, but cited lack of confidence to manage behavioural and cognitive difficulties (23). Interestingly, similar research exploring the attitudes of physiotherapy students found that 53% felt their academic training was sufficient to equip them with the skills to work with people with dementia (24), suggesting a reduction in confidence from undergraduate level to postgraduate level.

Our data suggests that the personal values, and thus the attitude, towards a person with advanced dementia was pivotal in determining the care that was delivered and received. Our participants were all specialists in treating people with advanced dementia and therefore had compassion and interest in treating this population, however they reported numerous examples where poor personal values of the HCP negatively affected the person with dementia – and thus their outcomes. Research has explored the importance of providing education and training for healthcare professionals relating to dementia in the acute setting (25), with a highlighted requisite that this education supports the development of empathy and understanding relating to the person with dementia (25, 26). However, much of this research focuses on attitudes in relation to knowledge, rather than the personal values of the HCP and how these influence the care provided.

The culture of the organisation was suggested to influence the HCPs values and conversely, HCPs with poor values relating to dementia could negatively affect the culture of the organisation. The culture of care has been linked to the value placed on the person with dementia (27), thus placing greater value on the person facilitates a culture which supports them. However, it has been suggested that education alone is not sufficient to facilitate this positive culture (25, 26). Organisations need a strong, positive leadership culture which promotes person centred care (28) and such leaders need to act as role models to demonstrate positive care. For people with complex needs, such as those with advanced dementia, situational leadership is recommended in the nursing literature (29) to support those with less experience. Lack of time and suitable environments were cited as being major barriers to facilitating effective interventions for people with advanced dementia and must be considered key to developing this positive culture.

Our data suggests that the inter-relationship between the knowledge and values of the HCP and the culture of the organisation are all key to successful outcomes. Our data (table 2) suggests that only positive attributes of all three domains will result in successful outcomes for people with advanced dementia.

Strengths and Limitations

For peer review only

This study used robust methods of data collection and analysis and followed clear guidance for undertaking such qualitative work. This included the use of COREQ (see supplementary material) to ensure clear and transparent methods and analysis. Although this was a small-scale qualitative study with a limited range of healthcare professionals taking part- which could impact on generalisability- sampling was deliberately specific and engaged HCPs who were specialists in treating people with very advanced dementia. This reduced the potential sample size, as there is a limited sample of people due to the specialist nature of interventions.

Conclusion

This qualitative study contributes to the knowledge about key domains to consider when providing rehabilitation interventions for people with advanced dementia. We propose that there are three key principles which must be optimised to ensure the best outcomes for people. The organisational culture should support person centred care approaches, while providing sufficient resources to support interventions. The HCP must have appropriate dementia specific knowledge and their personal values must align with the requirements for people with dementia. Should all key domains be optimised, the person with advanced dementia has potential to engage successfully with rehabilitation interventions. Further research is needed to determine the content of the actual interventions that are delivered; however we recommend that considerations are made to ensure that the person with dementia has the best chance of receiving meaningful rehabilitation.

For peer review only

Contributors

Conceptualization: AH.; methodology: AH, FM, VG; formal analysis: AH, FM, VG. ; data curation; AH, FM, VG..; writing—original draft: AH.; writing—review and editing: AH, FM, VG.; supervision: VG.; All authors have read and agreed to the published version of the manuscript.

Funding

This study is independent research funded by the National Institute for Health and Care Research (NIHR) School for Primary Care Research (119247) and supported by the NIHR Applied Research Collaboration South West Peninsula. The views expressed in this publication are those of the author(s) and not necessarily those of the National Institute for Health Research or the Department of Health and Social Care.

Ethics approval and consent to participate

The study was conducted according to the guidelines of the Declaration of Helsinki, and approved by the and ethical approval was obtained from the University of Exeter's College of Medicine and Health Research Ethics Committee (reference 515745). Informed consent was obtained from all subjects involved in the study.

Data Availability Statement

The datasets generated during and/or analyzed during the current study are available from the corresponding author upon reasonable request.

Acknowledgments

The authors thank all the participants who devoted their time to participate in this study.

Competing interests

The authors declare no conflict of interest or competing interests.

For peer review only

References

1. World Health Organisation. WHO global strategy on people-centred and integrated health services: interim report. World Health Organization; 2015.
2. World Health Organisation. Dementia: a public health priority: World Health Organization; 2012.
3. Ahmadi-Abhari S, Guzman-Castillo M, Bandosz P, Shipley MJ, Muniz-Terrera G, Singh-Manoux A, et al. Temporal trend in dementia incidence since 2002 and projections for prevalence in England and Wales to 2040: modelling study. bmj. 2017;358.
4. Mitchell SL. Advanced dementia. New England Journal of Medicine. 2015;372(26):2533-40.
5. Banerjee S, Smith S, Lamping D, Harwood R, Foley B, Smith P, et al. Quality of life in dementia: more than just cognition. An analysis of associations with quality of life in dementia. Journal of Neurology, Neurosurgery & Psychiatry. 2006;77(2):146-8.
6. Hall AJ, Febrey S, Goodwin VA. Physical interventions for people with more advanced dementia–a scoping review. BMC geriatrics. 2021;21(1):1-10.
7. Forbes D, Forbes S, Morgan DG, Markle-Reid M, Wood J, Culum I. Physical activity programs for persons with dementia. Cochrane Database of Systematic Reviews. 2008(3).

8. Lamb S, Mistry D, Alleyne S, Atherton N, Brown D, Copsey B, et al. Aerobic and strength training exercise programme for cognitive impairment in people with mild to moderate dementia: the DAPA RCT. Health Technology Assessment. 2018;22(28).
9. Blondell SJ, Hammersley-Mather R, Veerman JL. Does physical activity prevent cognitive decline and dementia?: A systematic review and meta-analysis of longitudinal studies. BMC public health. 2014;14(1):1-12.
10. Groot C, Hooghiemstra AM, Raijmakers PG, van Berckel BN, Scheltens P, Scherder EJ, et al. The effect of physical activity on cognitive function in patients with dementia: a meta-analysis of randomized control trials. Ageing research reviews. 2016;25:13-23.
11. Engel GL. The need for a new medical model: a challenge for biomedicine. Science. 1977;196(4286):129-36.
12. Goodwin VA, Allan LM. 'Mrs Smith has no rehab potential': does rehabilitation have a role in the management of people with dementia? Age and Ageing. 2018;48(1):5-7.
13. Cations M, May N, Crotty M, Low LF, Clemson L, Whitehead C, et al. Health Professional Perspectives on Rehabilitation for People With Dementia. Gerontologist. 2020;60(3):503-12.
14. Hall AJ, Fullam J, Lang IA, Endacott R, Goodwin VA. Community physiotherapy for people with dementia following hip fracture: Fact or fiction? A qualitative study. Dementia. 2020;19(8):2750-60.
15. Scerri A, Innes A, Scerri C. Healthcare professionals' perceived challenges and solutions when providing rehabilitation to persons living with dementia—A scoping review. Journal of Clinical Nursing.n/a(n/a).
16. Mitchell R, Fajardo Pulido D, Ryder T, Norton G, Brodaty H, Draper B, et al. Access to rehabilitation services for older adults living with dementia or in a residential aged care facility following a hip fracture: healthcare professionals' views. Disability and Rehabilitation. 2021;43(6):834-45.
17. McCarroll C, van't Riet C, Halter M. How is the emerging role of domiciliary physiotherapists who treat residents with dementia in nursing homes perceived by allied health professionals? A phenomenological interview study. Health & Social Care in the Community. 2020;28(1):279-90.
18. Borbasi S, Jones J, Lockwood C, Emden C. Health professionals' perspectives of providing care to people with dementia in the acute setting: Toward better practice. Geriatric Nursing. 2006;27(5):300-8.
19. Røsvik J, Rokstad AMM. What are the needs of people with dementia in acute hospital settings, and what interventions are made to meet these needs? A systematic integrative review of the literature. BMC Health Services Research. 2020;20(1):723.
20. Clarke V, Braun V. Thematic analysis. Encyclopedia of critical psychology: Springer; 2014. p. 1947-52.
21. Lynch C. World Alzheimer Report 2019: Attitudes to dementia, a global survey: Public health: Engaging people in ADRD research. Alzheimer's & Dementia. 2020;16:e038255.

For peer review only

22. Zhao W, Moyle W, Wu MLW, Petsky H. Hospital healthcare professionals' knowledge of dementia and attitudes towards dementia care: A cross-sectional study. Journal of Clinical Nursing. 2022;31(13-14):1786-99.
23. Hunter SW, Divine A. Understanding the factors influencing physiotherapists' attitudes towards working with people living with dementia. Physiotherapy Theory and Practice. 2021;37(12):1448-55.
24. Hunter SW, Armstrong J, Silva M, Divine A. Physiotherapy students' attitudes toward working with people with dementia: A cross-sectional survey. Physical & Occupational Therapy In Geriatrics. 2020;38(1):56-66.
25. Cowdell F. Care of older people with dementia in an acute hospital setting. Nursing Standard (through 2013). 2010;24(23):42.
26. Moyle W, Murfield JE, Griffiths SG, Venturato L. Care staff attitudes and experiences of working with older people with dementia. Australasian Journal on Ageing. 2011;30(4):186-90.
27. Houghton C, Murphy K, Brooker D, Casey D. Healthcare staffs' experiences and perceptions of caring for people with dementia in the acute setting: Qualitative evidence synthesis. International Journal of Nursing Studies. 2016;61:104-16.
28. Rokstad AMM, Vatne S, Engedal K, Selbæk G. The role of leadership in the implementation of person-centred care using Dementia Care Mapping: a study in three nursing homes. Journal of Nursing Management. 2015;23(1):15-26.
29. Lynch BM, McCormack B, McCance T. Development of a model of situational leadership in residential care for older people. Journal of Nursing Management. 2011;19(8):1058-69.

For peer review only

COREQ (COnsolidated criteria for REporting Qualitative research) Checklist

A checklist of items that should be included in reports of qualitative research. You must report the page number in your manuscript where you consider each of the items listed in this checklist. If you have not included this information, either revise your manuscript accordingly before submitting or note N/A.

Topic	Item No.	Guide Questions/Description	Reported on Page No.
**Domain 1: Research team and reflexivity**			
Personal characteristics			
Interviewer/facilitator	1	Which author/s conducted the interview or focus group?	
Credentials	2	What were the researcher's credentials? E.g. PhD, MD	
Occupation	3	What was their occupation at the time of the study?	
Gender	4	Was the researcher male or female?	
Experience and training	5	What experience or training did the researcher have?	
Relationship with participants			
Relationship established	6	Was a relationship established prior to study commencement?	
Participant knowledge of the interviewer	7	What did the participants know about the researcher? e.g. personal goals, reasons for doing the research	
Interviewer characteristics	8	What characteristics were reported about the inter viewer/facilitator? e.g. Bias, assumptions, reasons and interests in the research topic	
**Domain 2: Study design**			
Theoretical framework			
Methodological orientation and Theory	9	What methodological orientation was stated to underpin the study? e.g. grounded theory, discourse analysis, ethnography, phenomenology, content analysis	
Participant selection			
Sampling	10	How were participants selected? e.g. purposive, convenience, consecutive, snowball	
Method of approach	11	How were participants approached? e.g. face-to-face, telephone, mail, email	
Sample size	12	How many participants were in the study?	
Non-participation	13	How many people refused to participate or dropped out? Reasons?	
Setting			
Setting of data collection	14	Where was the data collected? e.g. home, clinic, workplace	
Presence of non-participants	15	Was anyone else present besides the participants and researchers?	
Description of sample	16	What are the important characteristics of the sample? e.g. demographic data, date	
Data collection			
Interview guide	17	Were questions, prompts, guides provided by the authors? Was it pilot tested?	
Repeat interviews	18	Were repeat inter views carried out? If yes, how many?	
Audio/visual recording	19	Did the research use audio or visual recording to collect the data?	
Field notes	20	Were field notes made during and/or after the inter view or focus group?	
Duration	21	What was the duration of the inter views or focus group?	
Data saturation	22	Was data saturation discussed?	
Transcripts returned	23	Were transcripts returned to participants for comment and/or	

For peer review only

Topic	Item No.	Guide Questions/Description	Reported on Page No.
		correction?	
**Domain 3: analysis and findings**			
Data analysis			
Number of data coders	24	How many data coders coded the data?	
Description of the coding tree	25	Did authors provide a description of the coding tree?	
Derivation of themes	26	Were themes identified in advance or derived from the data?	
Software	27	What software, if applicable, was used to manage the data?	
Participant checking	28	Did participants provide feedback on the findings?	
Reporting			
Quotations presented	29	Were participant quotations presented to illustrate the themes/findings? Was each quotation identified? e.g. participant number	
Data and findings consistent	30	Was there consistency between the data presented and the findings?	
Clarity of major themes	31	Were major themes clearly presented in the findings?	
Clarity of minor themes	32	Is there a description of diverse cases or discussion of minor themes?	

Developed from: Tong A, Sainsbury P, Craig J. Consolidated criteria for reporting qualitative research (COREQ): a 32-item checklist for interviews and focus groups. *International Journal for Quality in Health Care*. 2007. Volume 19, Number 6: pp. 349 – 357

Once you have completed this checklist, please save a copy and upload it as part of your submission. DO NOT include this checklist as part of the main manuscript document. It must be uploaded as a separate file.

For peer review only

TOPIC GUIDE

Researcher to introduce self and explain process of focus group, group rules, confidentiality, permission to record, right to withdraw.

Topic	Prompts
**Introduction – each member**<br><br>Please can you tell me a bit about your experience of working with people with advanced dementia and any challenges that you experience supporting them	Profession<br><br>Interest in PwD<br><br>System/organisational pressures<br><br>Individual patient difficulties<br><br>Carer
**Treatments**<br><br>What things are important to ensure that a treatment with a person with advanced dementia is effective?	Who present?<br><br>Involvement of family/carers<br><br>Duration<br><br>Type of intervention<br><br>Patient outcomes / QoL<br><br>Daily routines
**Location**<br><br>What are the key considerations when providing healthcare interventions in a nursing home compared to at home?	Environment<br><br>Formal carers – engagement, education, knowledge<br><br>Equipment
**Communication**<br><br>What factors are important to consider when communicating with a person with advanced dementia?	Body language<br><br>Tone

For peer review only

	Language
**Training/education**<br><br>Therapists with a variety of experience will be treating people with advanced dementia, therefore what education or skills are needed to be able to treat people with advanced dementia?	Undergraduate learning<br><br>Formal learning<br><br>Experiential learning

Is there something else that I have not asked you about, that you would like to tell me about?

Thank you

For peer review only

BMJ Open

A qualitative study exploring health care professionals' perceptions of providing rehabilitation for people with advanced dementia

Journal:	*BMJ Open*
Manuscript ID	bmjopen-2023-072432.R3
Article Type:	Original research
Date Submitted by the Author:	14-Jul-2023
Complete List of Authors:	Hall, Abigail J.; University of Exeter, Public Health and Sports Science<br>Manning, Fay; University of Exeter Medical School, Department of Medical Imaging<br>Goodwin, Victoria; University of Exeter, Public Health and Sports Science
**Primary Subject Heading**:	Rehabilitation medicine
Secondary Subject Heading:	Qualitative research
Keywords:	Dementia, REHABILITATION MEDICINE, QUALITATIVE RESEARCH

SCHOLARONE™
Manuscripts

I, the Submitting Author has the right to grant and does grant on behalf of all authors of the Work (as defined in the below author licence), an exclusive licence and/or a non-exclusive licence for contributions from authors who are: i) UK Crown employees; ii) where BMJ has agreed a CC-BY licence shall apply, and/or iii) in accordance with the terms applicable for US Federal Government officers or employees acting as part of their official duties; on a worldwide, perpetual, irrevocable, royalty-free basis to BMJ Publishing Group Ltd ("BMJ") its licensees and where the relevant Journal is co-owned by BMJ to the co-owners of the Journal, to publish the Work in this journal and any other BMJ products and to exploit all rights, as set out in our licence.

The Submitting Author accepts and understands that any supply made under these terms is made by BMJ to the Submitting Author unless you are acting as an employee on behalf of your employer or a postgraduate student of an affiliated institution which is paying any applicable article publishing charge ("APC") for Open Access articles. Where the Submitting Author wishes to make the Work available on an Open Access basis (and intends to pay the relevant APC), the terms of reuse of such Open Access shall be governed by a Creative Commons licence – details of these licences and which Creative Commons licence will apply to this Work are set out in our licence referred to above.

Other than as permitted in any relevant BMJ Author's Self Archiving Policies, I confirm this Work has not been accepted for publication elsewhere, is not being considered for publication elsewhere and does not duplicate material already published. I confirm all authors consent to publication of this Work and authorise the granting of this licence.

For peer review only

A qualitative study exploring health care professionals' perceptions of providing rehabilitation for people with advanced dementia

Abigail J Hall BSc, MSc, PhD, MCSP

Public Health and Sports Science, University of Exeter, Heavitree Road, Exeter, EX1 2LU, UK

a.hall4@exeter.ac.uk

Fay Manning BSc, MSc, PhD

Department of Medical Imaging, University of Exeter, Heavitree Road, Exeter, EX1 2LU, UK

Victoria A Goodwin, MBE, BSc, MSc, PhD, FCSP,

Public Health and Sports Science, University of Exeter, Heavitree Road, Exeter, EX1 2LU, UK

Correspondence to:

Abigail J Hall

Public Health and Sports Science, University of Exeter, Heavitree Road, Exeter, EX1 2LU, UK

a.hall4@exeter.ac.uk

For peer review only

Abstract

Objectives

The aim of this study was to explore health care professionals' principles for providing and delivering rehabilitation interventions for people with advanced dementia.

Design

This was a qualitative study with three focus groups undertaken virtually. The data was analysed using a process of reflexive thematic analysis in order to gain an in depth understanding of rehabilitation principles for this population.

Setting and participants

20 healthcare professionals who were specialists in treating and rehabilitating people with advanced dementia were recruited. These healthcare professionals had a wide range of experience in a variety of different settings including primary care, secondary care as well as specialist mental health teams. Purposive sampling focused on the requirement for participants to have significant experience of treating people with dementia. Participants were from the UK and Denmark. Data collection was undertaken during August and September 2022.

Results

Three overarching themes were developed following analysis – organisational culture, knowledge and personal values of the health care professional. The first explored how the culture of an organisation affects a person with advanced dementia as well as the healthcare professional. The organisation needed to promote positive approaches to person centred care and provide effective situational leadership to embed such approaches. Knowledge was a key consideration and was closely linked to the personal values of the healthcare professional, which formed the final theme.

For peer review only

This study suggests that the interrelationship of these three factors influences the outcomes for the person with dementia and effective outcomes required consideration of all domains.

Conclusions

Effective interventions for people with advanced dementia require the healthcare professional to have the knowledge about dementia and positive personal values, but the culture of the organisation is also key to ensure that the healthcare professional is able to deliver successful interventions.

Article summary

Strengths and limitations of this study

- This work was informed and supported by an active Patient Participation Involvement group—including collaborating with designing the study and discussing the findings.
- The methods of data collection and analysis were robust and followed clear guidance for undertaking such qualitative work.
- The study was reported using COREQ guidelines to ensure clear and transparent methods and analysis.
- Although this was a small-scale qualitative study with a limited range of healthcare professionals taking part, which could impact on generalisability, sampling was deliberately specific and engaged healthcare professionals who were specialists in treating people with very advanced dementia.

Introduction

Dementia is a neurological disorder resulting in physical and cognitive decline, found typically in older adults (1, 2). It is estimated that by 2040, there will be 1.2 million people with dementia living in the UK – a 57% increase from 2016 (3), suggesting that dementia will become a national – and global – challenge to healthcare services. Advanced dementia is characterised by profound communication, memory and physical disturbances (4), which often result in significant impairments in a person's ability to undertake activities of daily living and a perceived decline in quality of life (5).

A variety of healthcare professionals (HCPs) can be involved in trying to reduce the physical declines associated with advanced dementia, including physiotherapists and occupational therapists. However, there remains little evidence to support physical interventions for people with dementia (6-10). This lack of evidence is particularly evident for people with more severe dementia, where physical interventions to improve physical ability – or reduce the speed of decline – are largely not understood (6).

There is much debate about the approach that is suitable for people with advanced dementia, with greater emphasis being placed on adopting a biopsychosocial model, where functioning and participation is key. Some 60 years ago, a key paper - "The need for a new model: A challenge for biomedicine" (11) argued that the dominant biomedical model of disease was insufficient for a

For peer review only

complete understanding of health. It was stated that a biomedical approach ''*assumes disease to be fully accounted for by deviations from the norm of measurable, biological variables. It leaves no room within its framework for the social, psychological and behavioural dimensions of illness''* (p. 130). Considering not only biological components, but also the individual and societal contexts of the individual's experiences is vital in the rehabilitation of people living with dementia to ensure a person-centred approach to achieve what is meaningful to the individual. However, the challenge that this poses to healthcare professionals is poorly understood – especially for people living with more advanced dementia (6, 12-14).

The experience of health care professionals treating people with dementia has been explored in a variety of settings (15, 16), including residential aged care facilities (16, 17) and acute settings (18). A recent review exploring the experiences of caring for people with dementia in acute settings has highlighted the importance of developing knowledge of person centred approaches to care, however research has also highlighted a lack of understanding about the importance of the environment and how this may impact care (19). However, evidence suggests that providing rehabilitation interventions for people with more advanced dementia is different to those with more mild forms (6, 14). Thus, the aims of the study were to explore the rehabilitation principles of HCPs providing and delivering rehabilitation interventions for people with advanced dementia in a variety of settings including acute, community and residential care.

Methods

Design

During August and September 2022, three semi-structured focus groups were undertaken with a range of HCPs. These health professionals were all specialists in the management or rehabilitation of people living with advanced dementia. The focus groups were undertaken virtually to reduce barriers due to geographical limitations and enabled international specialists to contribute. The study has been reported according to the COREQ guidelines (Supplementary material 1).

Setting and participants

A purposive sampling strategy was employed to seek HCPs who specialise in working with people with advanced dementia. A targeted social media advert sought professionals with advanced skills and pre-existing networks were drawn upon to recruit participants. Some of these participants were known to the researchers in a professional capacity due to previous work undertaken in this area. This did not affect the data collection.

Inclusion criteria: specialist working with people with dementia as a physiotherapist, occupational therapist, psychologist, nurse, or other related profession. ("Specialist" refers to the level of experience and training that an HCP has and an expectation that they are working in an advanced role relating to the care of people with dementia)

Participants who responded to the social media advert or initial email were sent a follow up email with the participant information sheet attached. At this point they were offered an opportunity to

For peer review only

discuss the study further if they had any particular questions. If they were happy to take part in the study, they were asked to provide informed consent prior to the commencement of the focus group. Participants were informed that they were under no obligation to participate in the focus group and they may withdraw from it at any time, without any negative consequence, up until the point where the data were fully anonymised.

Provided the participant met the inclusion criteria, all participants who consented to take part were invited to join a focus group. Recruitment continued until sufficient data was obtained to be able to answer the aim of the study and no new data was being generated.

Data collection

The focus groups aimed to explore the challenges and techniques required to effectively manage people with more advanced dementia, while also considering the principles of rehabilitation for this population. The focus groups were facilitated by two experienced qualitative researchers (AH and FM). FM acted as a 'second facilitator/observer', taking notes and making observations pertaining to interaction of the group. Participants were all encouraged to give their opinions and thoughts during the meeting. To ensure that everybody was given the opportunity to contribute, each participant was asked whether they had further detail to add to any of the topics before moving on to the next. Both researchers have undertaken training courses pertaining to qualitative analysis and have published multiple qualitative papers.

Microsoft Teams was used to undertake the focus groups and they were recorded and automatically transcribed. The transcription function has been used by the researcher and found to be accurate, although the transcripts were all checked for accuracy. The focus groups lasted for a maximum of 90 minutes and included time for participants to introduce themselves and their roles to other members of the group. The participants were informed that this study formed part of a larger piece of work aiming to develop interventions for people with advanced dementia. A semi-structured guide (Supplementary material 2) was used to guide the conversation of the focus groups. The topic guide was not piloted prior to the initial focus group as it was anticipated that it could be adapted for subsequent focus groups if needed. This was not required and the same topic guide was used for all interviews. The data was anonymised so that individual participants could not be identified, and all participants consented to their data being transcribed and analysed. Transcripts were not reviewed by participants. The data collection from the focus groups provided rich and detailed data and no further follow-up interviews were deemed necessary although people were offered the opportunity to discuss any further points following completion of the focus group.

Patient and public involvement

A patient and public involvement (PPI) group was developed to help inform the research undertaken during this study and as part of a wider programme of work. The group has been evolving and developing and now consists of people with a variety of experiences of dementia. The group includes people living with dementia, carers of people with dementia and two health care professional who have personal experience of dementia.

For peer review only

Initial discussions focused on the challenges they had experienced with getting rehabilitation for people who have dementia – especially when the person had advanced dementia or was living in a care home. Several themes generated from the PPI members formed part of the focus group discussion. Due to practical challenges, none of the PPI group were able to participate in the focus groups, but discussion was held with representatives following the groups and helped guide the analysis and results.

Analysis

Analysis commenced after the first focus group was completed and continued throughout the following focus groups. As the data collection and initial analysis ran simultaneously, arising themes or gaps could be probed accordingly in the remaining focus groups.

NVivo 11 (QSR International) was used to organize and code the data. This facilitated a process of reflexive thematic analysis to be undertaken - as suggested by Braun and Clarke (20). Familiarisation of the data involved reading and re-reading the transcripts. A process of systematic open coding was then commenced and initial ideas for themes were noted. Following this open coding, initial themes were generated, discussed and explored with the wider research team. These discussions led to further refinement of the themes, and definitions written to ensure clarity between the research team. For example, there were multiple codes relating to participants using positive success stories to educate others, this was collated into a minor theme of "optimism/wisdom" which was then classified within the "personal values of the HCP". This process was repeated for all the data until themes were developed. This process was an iterative process and resulted in several refinements of named themes. These themes were then discussed with the patient and public involvement group who were supporting and collaborating on the overarching project.

Ethics

Ethical approval was obtained from the University of Exeter's College of Medicine and Health Research Ethics Committee (reference 515745).

Findings

20 healthcare professionals took part in three focus groups (Table 1). Only one person who had initially expressed an interest in taking part did not reply to the meeting invite and therefore did not take part. Following the third focus group, it was felt that no new themes were occurring and therefore it was believed that theoretical data saturation had been reached and no further focus groups were planned. There were no withdrawals from the study at any stages. The HCPs reported an average of eighteen years of experience and an average of thirteen years specializing specifically with people with dementia. The settings of their experience were broad, including in-patient settings, out-patient clinics, patients' own homes and residential/nursing settings.

For peer review only

Table 1. Participant characteristics (n=20)

**Sex**	**Female**	**19**
	Male	1
**Profession**	Physiotherapist	14
	Occupational Therapist	3
	Nurse	2
	Psychologist	1
**NHS Grade / Banding**	6	6
	7	8
	8+	2
	N/A	4
**Years of clinical experience**	0-5	1
	6 – 10	2
	11-15	2
	16 – 20	3
	21 – 25	6
	26 – 30	2
	30+	3
	Not reported	1
**Years of experience working with people with advanced dementia**		
	0-5	4
	6 – 10	2
	11-15	6
	16 – 20	5
	21 – 25	2
	26 – 30	1
	30+	0
**Location**	England	13
	Wales	2
	Scotland	4
	Denmark	1
**% of caseload of people with dementia**		
	0 – 25	1
	26 – 50	1
	51 – 75	6
	76 – 100	8
	Not currently working clinically	4
**Locations of work**	In-patient	12
	Out-patient	4
	Care home/residential homes	11
	Own homes	11
	Other	2

For peer review only

The principles of rehabilitation were explored for people with advanced dementia and three overarching themes were developed following analysis. These were 'organisational culture', 'knowledge about dementia' and 'personal values of the HCPs'. The first theme explores how organisational culture may affect the person with advanced dementia as well as the HCP working within a system or organisation. Secondly, knowledge of dementia was considered to be a key consideration when exploring the rehabilitation of people with advanced dementia, but this was closely linked to the personal values of the healthcare professional, which formed the final theme. Each theme will be explored in further detail and then the interaction between them considered.

Organisational culture

Our participants discussed how there was a varied understanding of what "rehabilitation" means to people and how this differing understanding led to people with advanced dementia receiving a wide variety of interventions. This often related to understanding what the purpose of rehabilitation was, from seeking to gain functional improvements to preventing decline. There was also discussion about the consideration of how services and organisations' cultures affected the interventions that were delivered.

Determining what the term "rehabilitation" means was reported to be challenging for healthcare professionals generally. What an organisation considered to be rehabilitation would significantly affect what was delivered. Our participants were very clear that for people with advanced dementia, tasks that were perceived to be 'simple' were actually very important and, although not deemed as "traditional rehabilitation" were actually important aspects of rehabilitation.

> *"we talk a lot in our training about the concept of rehab as what we find is if.... they're not looking to get them back up on their feet, then that's not rehab. Whereas my argument that is actually for somebody with quite an advanced dementia, we have help them into a sitting position, can have massive functional benefits for them. So that in itself is rehab" (PT9)*

The term "traditional rehabilitation" was discussed in all focus groups and led back to the underlying biomedical principles that historically have been adopted by many healthcare professionals. This approach was focused on the injury or illness.

> *".....[we] know all about anatomy and physiology and this is how to treat a broken leg. This is how to treat a back pain. But they don't actually get taught how to embrace that full person centered approach to things." (PT7)*

There was also a noted discomfort that HCPs felt that they had to justify doing things differently – and thus outcome measures were described as being vital to "prove effectiveness" of their interventions. Often organisations demanded these outcome measures to prove their services were effective. It was also discussed that often it was only the physical interventions that were perceived to be treatment – to such an extent that only the physical interventions were often documented. Our participants were very clear that these "non-traditional" approaches needed to be clearly documented to justify their interventions.

For peer review only

"I think is well, it's about how we document that because we don't always document those times when we've gone in and not been able to engage with somebody. We maybe say they weren't able to engage in our documentation, but we don't say we spent time engaging with that person and it resulted in an improved rapport, improved connection." (PT7)

Complexity of patients was noted as requiring a different approach and people with advanced dementia were recognised as being highly complex and therefore needing care to be adapted accordingly.

"We're trying to pretend that we haven't got a complex system….which a complex patient is…. we're still trying to imagine that we've got a simple machine where if you put one thing in change, one of the dials, you're gonna get a completely different product out. And so it goes right back to undergraduate education and absolutely rethinking our model of physiotherapy." (PT1)

It was discussed that the focus of rehabilitation has often been to make a tangible improvement after an illness or injury which initially resulted in a decline in physical function. However, our participants questioned this and reported that often for people with advanced dementia, rehabilitation could be considered a slowing of the inevitable physical decline that is associated with the condition.

"….because it's a degenerative disorder and …. the processes that these patients will deteriorate…..it's not just about improving things for them, but maintaining what they're able to do for as long as physically possible as well" (PT6)

While there was a focus on preventing decline, there was also belief that people with advanced dementia can improve physically – therefore this should be a focus of the intervention. However, it was clear that this potential to improve physically required timely – and appropriately targeted – interventions, often before there was significant physical decline.

The unclear nature of interventions for people with advanced dementia makes it difficult to determine their trajectory of physical recovery, however, our HCPs felt very strongly that people needed to be given an opportunity to receive rehabilitation. In the acute setting, giving these opportunities was challenging due to the focus on discharging patients to ensure good patient "flow". Despite this challenge, our participants were determined to offer as much as they possibly could, but they recognised this was often insufficient.

"Yeah, time, time for us… how long you can spend with the patient. Obviously you know you have so many patients to split your time between. But our biggest time pressure is discharge pressures." (PT13)

The influence of the service or organisation that a person was working within had a significant impact on the interventions that could be delivered. The way that an organisation did, or in some cases did not, adapt their environments and policies to suit a person with advanced dementia would influence their journeys.

"The acute sector is massively, massively limited in what it can provide people with dementia…. the environment is completely wrong. It's wrong for people

without dementia. It's completely wrong for people with dementia, so of course they're not gonna give their best selves." (PT4)

While the acute setting had a significant focus on patient flow, the actual environment was deemed very unsuitable for people with advanced dementia. Noise, inappropriate lighting and distractions would often add to challenging behaviours that the person might display. Organisations needed to ensure their environments were suitable.

"we do seem to have a bit of an issue, I call it beige bays, we create these spaces and we set people in them and we say 'now you stay there for six hours, 10 hours, 12 hours and only get up if you need to go to the toilet'. We….actively deprive people of activity during their stay and then get really shocked why they start getting really curious/bored out of their brains and wanting to get up and moving." (OT2)

Organisations often were risk averse and fear of people falling and injuring themselves led to risk averse behaviours such as discouraging mobility or keeping people in bed, but HCPs feared this and actively tried to overcome these challenges. Discouraging activity led to deconditioning and deterioration of function which was considered a significant risk of harm in itself.

"I don't like the term deconditioning, because I think it's completely poor care and lack of facilitating to function… We've allowed this this appalling situation to happen where when our older people go into hospital, they deteriorate almost in front of in fact definitely in front of our eyes." (PT1)

There was a frustration that often people with advanced dementia were restricted from undertaking "risky" activities, but there was little emphasis placed on how this would affect the person from an emotional perspective.

"I hear people talking about the talking about the risk of falling over the risk of pressure ulcers, that risk of infection. We have huge risks to personhood, to selfhood, to self esteem, to independence, to the ability to live independently." (PT1)

Our HCPs also reported frustration with arbitrary timescales that patients were given to receive an intervention. Whether this was due to waiting lists for community services which were reported to be up to 40 weeks in some services, to the time limited services such as in-patient intermediate care or rehabilitation.

"….if you're gonna give people time, you need to look at the whole system, that of rehab….who came up with six weeks? I've got no idea. But that seems to be "if you can't get it and six weeks, then there's no point really" attitude." (PT9)

Knowledge relating to dementia

For peer review only

Knowledge relating to dementia and a good understanding of person centred care was deemed vital to be able to effectively treat a person with advanced dementia. Where there was a lack of knowledge, there were often poor outcomes for the person.

Knowledge relating to dementia was seen as being key to providing effective interventions. Our HCPs described challenges in gaining this knowledge, with many relying on experience and learning "on the job". Undergraduate education for HCPs was universally felt to be inadequate and therefore, postgraduate education was vital, although difficult to access.

> *"The standard of university education on dementia for physios in particular and frailty is really, really poor and there's a lot and a lot of education that us more experienced physios need to be doing…. and that's why the words "no rehab potential" ….[are] still out there. Because there's not a lot of education around frailty and older people" (PT4)*

HCPs working in mental health settings found access to specific mental health training more accessible generally, although this was often generic rather than profession specific – this was particularly reported by physiotherapists who reported a lack of physiotherapy specific dementia training. There was a universal feeling amongst our participants that a large element of their role was about teaching and educating others – about dementia itself, but also the importance of delivering person centred care approaches to interventions. This related to other HCPs, but also to patients themselves and their carers.

A lack of knowledge and understanding was deemed to precipitate the belief that people with dementia had "no rehabilitation potential". Indeed, this term was reported to be used very commonly.

> *"the staff who are labelling individuals of "no rehab potential" are often the less experienced staff ….. making a snapshot sort of decision within 10 minutes or five minutes." (PT2)*

Person centred approaches to care were discussed in relation to a person with advanced dementia, but also in terms of how treatments should be adapted and personalised for everybody.

> *"It doesn't really matter if a patient has advanced dementia or if they're me, a 28 year old that's had knee surgery. If an exercise makes no sense to me, or if it's boring, or if it's not gonna help me get back to what I want to do, I'm probably not gonna do it. And really, if we make healthcare better for those with advanced dementia, we're gonna make it better for everyone, because what we're talking about there is personalized care, isn't it?" (PT4)*

This emphasis on ensuring that interventions were tailored to individuals was reported to be even more important to a person with advanced dementia, but understanding how to tailor interventions was variable and influenced by external factors such as time and availability of resources.

> *"…that time pressure because I think patients feel it and I think I might, you know, [allied health profession] colleagues feel it as well because literally as soon as people are either hitting a rehab ward or getting into an [intermediate care] bed,*

For peer review only

> *you're looking at discharge planning before you've even really got to the crux of that rehab" (PT9)*

Our HCPs reported that people were very aware of the concepts of person-centred approaches to care, but these were often seen as gold standard and their services were not always deemed able to achieve this. Nor did they always have a good understanding about how to actually deliver such approaches. There was also suggestion that person centred care was not taught at undergraduate level in some professional groups, and this was something that had to be learnt as a post-graduate.

Personal values of the HCP

It was evident that the personal values of HCPs providing care for people with advanced dementia could significantly impact on the delivery of that care.

An interest in, and taking time, to get to know a person was deemed vital to be able to provide interventions to people with advanced dementia. However, a natural curiosity was evident amongst our participants. Where little formal education had been received, their curiosity to discover "what works" led them to a particular interest and passion for treating people with dementia. However, this was deemed to be the exception rather than the rule and many of our participants reported feeling like a "*lone voice*" where few other people had an interest in this population.

> *"And the other point I just want to make is sometimes you feel like a lone voice out here kind of shouting about how important these things are. And I feel like I'm not on my own for a while." (PT7)*

However, not all HCPs had this level of curiosity or interest about working with people with advanced dementia. Many of our participants provided teaching and education to others relating to dementia, but often with little positive engagement.

> *"I actually got onto the physio course for them for me to teach them and I did two consecutive years …..People weren't interested….. a room of 50 people just shut down, as if cognition and old and people with cognitive frailty was something they'd never come across." (PT1)*

This was particularly reported amongst physiotherapists and there was a belief that occupational therapists were more involved in treating people with dementia or cognitive difficulties historically, although there was a feeling that this attitude was beginning to change. There was further evidence to support this with our participants discussing the role of physiotherapists in mental health teams being much less advanced than the role of occupational therapists.

> *"I was one of the first physiotherapist who was hired to work in a nursing home and in [country]…..I've worked a lot with nurses and caregivers and they've been really interested in what I could offer. I have [tried with] the physiotherapist. They weren't interested." (PT5)*

There was considerable optimism amongst our participants that people with advanced dementia could effectively receive rehabilitation and other interventions, but this came from positive previous

For peer review only

experience – their wisdom. It was notable that sharing this experience and positive outcomes was used as a way to engage other HCPs in treating people with advanced dementia.

Knowledge of negative outcomes for people with dementia also led dementia specialist HCPs to be more proactive in their approaches, often actively seeking out patients who would benefit from specialist interventions.

"….we've also changed our practice. Whenever anyone go to the to that trust, we've phoned and we make sure and we may actually go out." (PT10)

However, this optimism was often diminished by challenges relating to service pressures and limitation that the HCPs had to work within. Unsuitable ward environments were a particular challenge and the HCPs accepted the fact that the person with advanced dementia was unlikely to be able to improve in that setting and therefore focused their attention on ensuring they could be discharged to the most appropriate location to facilitate rehabilitation. Where this optimism was reduced, HCPs noted feeling very alone and isolated when trying to "fight" for people with dementia.

It was evident that our participants felt a significant amount of compassion towards their patients. There was discussion about the mutual upset that they felt when people with dementia received sub-optimal care, and this was clearly upsetting to participants – some giving examples of poor care that they had experienced. There was a belief that people with advanced dementia were often stigmatised and labelled, which prevented them from receiving the care that they needed and deserved.

"…there's this been this historic thing about 'no rehab potential'. And the consistent negative labelling of our people that we work with…….'No capacity, no rehab potential behaviours that challenge aggressive.' That that's always been the negative, negative and pejorative labelling." (PT1)

There was also a feeling of guilt and frustration where an HCP was not able to provide the intervention they wanted to – usually due to resource pressures or the culture of the organisation. However, it was evident that the individuals' attitude was key and their beliefs and engagement with people with advanced dementia had a pivotal role in determining what intervention was delivered.

"….attitude is huge. I think if you if you don't have the right attitude, nothing really follows….. three words that stand out for me are time, connection and value. So value in the person value, in the carer value, in their journey and where they've got to until now." (PT7)

The interrelationship between the values of the HCP, the organisational culture and the knowledge of the HCP is detailed in Figure 1. Our data suggests that in order for the person with dementia to have positive outcomes, all three variables need to be optimised.

Discussion

The aim of the study was to explore the rehabilitation principles when providing and delivering rehabilitation for people with advanced dementia. Our participants described the key aspects to

For peer review only

consider the culture of rehabilitation and raised questions about what this means, but also what other people and HCPs perceive it to be.

Our data suggests an important relationship between these factors and for a person with advanced dementia to receive optimal interventions there needs to be a symbiotic relationship between all three factors (Figure 1). Where any one of the elements is missing, the person with dementia will not receive the care that they need. However, there was a noted importance on the values of the HCP themselves and how these values could be directly influenced by the culture of the organisation and the knowledge about how best to manage people with advanced dementia.

There was a common belief that knowledge about treating people with advanced dementia was key and where there was a lack of knowledge outcomes were poor – or people simply weren't offered interventions. This knowledge related both to the pathogenesis of dementia, how to provide care for the person, but also how interventions could be suitably tailored to a accommodate the persons' cognitive abilities.

Knowledge about dementia amongst healthcare professionals is inconsistent, with one large study undertaken by Alzheimer's Disease International suggesting that 62% of healthcare professionals believed that dementia is a natural part of ageing and supported other studies findings that there was nothing that could be done to manage it (21, 22). However, it is unclear whether, and how, this relates to specific professions. Our data suggests a lack of undergraduate training for physiotherapists relating to dementia. A Canadian study surveyed physiotherapists and found that levels of knowledge relating to dementia was high, but cited lack of confidence to manage behavioural and cognitive difficulties (23). Interestingly, similar research exploring the attitudes of physiotherapy students found that 53% felt their academic training was sufficient to equip them with the skills to work with people with dementia (24), suggesting a reduction in confidence from undergraduate level to postgraduate level.

Our data suggests that the personal values, and thus the attitude, towards a person with advanced dementia was pivotal in determining the care that was delivered and received. Our participants were all specialists in treating people with advanced dementia and therefore had compassion and interest in treating this population, however they reported numerous examples where poor personal values of the HCP negatively affected the person with dementia – and thus their outcomes. Research has explored the importance of providing education and training for healthcare professionals relating to dementia in the acute setting (25), with a highlighted requisite that this education supports the development of empathy and understanding relating to the person with dementia (25, 26). However, much of this research focuses on attitudes in relation to knowledge, rather than the personal values of the HCP and how these influence the care provided.

The culture of the organisation was suggested to influence the HCPs values and conversely, HCPs with poor values relating to dementia could negatively affect the culture of the organisation. The culture of care has been linked to the value placed on the person with dementia (27), thus placing greater value on the person facilitates a culture which supports them. However, it has been suggested that education alone is not sufficient to facilitate this positive culture (25, 26). Organisations need a strong, positive leadership culture which promotes person centred care (28) and such leaders need to act as role models to demonstrate positive care. For people with complex needs, such as those with advanced dementia, situational leadership is recommended in the nursing

For peer review only

literature (29) to support those with less experience. Lack of time and suitable environments were cited as being major barriers to facilitating effective interventions for people with advanced dementia and must be considered key to developing this positive culture.

Our data suggests that the inter-relationship between the knowledge and values of the HCP and the culture of the organisation are all key to successful outcomes. Our data (Figure 1) suggests that only positive attributes of all three domains will result in successful outcomes for people with advanced dementia.

This study used robust methods of data collection and analysis and followed clear guidance for undertaking such qualitative work. This included the use of COREQ (Supplementary material 1) to ensure clear and transparent methods and analysis. Although this was a small-scale qualitative study with a limited range of healthcare professionals taking part- which could impact on generalisability- sampling was deliberately specific and engaged HCPs who were specialists in treating people with very advanced dementia. This reduced the potential sample size, as there is a limited sample of people due to the specialist nature of interventions.

Conclusion

This qualitative study contributes to the knowledge about key domains to consider when providing rehabilitation interventions for people with advanced dementia. We propose that there are three key principles which must be optimised to ensure the best outcomes for people. The organisational culture should support person centred care approaches, while providing sufficient resources to support interventions. The HCP must have appropriate dementia specific knowledge and their personal values must align with the requirements for people with dementia. Should all key domains be optimised, the person with advanced dementia has potential to engage successfully with rehabilitation interventions. Further research is needed to determine the content of the actual interventions that are delivered; however, we recommend that considerations are made to ensure that the person with dementia has the best chance of receiving meaningful rehabilitation.

For peer review only

Contributors

Conceptualization: AH.; methodology: AH, FM, VG; formal analysis: AH, FM, VG. ; data curation; AH, FM, VG..; writing—original draft: AH.; writing—review and editing: AH, FM, VG.; supervision: VG.; All authors have read and agreed to the published version of the manuscript.

Funding

This study is independent research funded by the National Institute for Health and Care Research (NIHR) School for Primary Care Research (119247) and supported by the NIHR Applied Research

Collaboration South West Peninsula. The views expressed in this publication are those of the author(s) and not necessarily those of the National Institute for Health Research or the Department of Health and Social Care.

Ethics approval and consent to participate

The study was conducted according to the guidelines of the Declaration of Helsinki, and approved by the and ethical approval was obtained from the University of Exeter's College of Medicine and Health Research Ethics Committee (reference 515745). Informed consent was obtained from all subjects involved in the study.

For peer review only

Data availability statement

The datasets generated during and/or analyzed during the current study are available from the corresponding author upon reasonable request.

Acknowledgements

The authors thank all the participants who devoted their time to participate in this study.

Competing interests

The authors declare no conflicts of interest or competing interests.

References

1. World Health Organisation. WHO global strategy on people-centred and integrated health services: interim report. World Health Organization; 2015.
2. World Health Organisation. Dementia: a public health priority: World Health Organization; 2012.
3. Ahmadi-Abhari S, Guzman-Castillo M, Bandosz P, Shipley MJ, Muniz-Terrera G, Singh-Manoux A, et al. Temporal trend in dementia incidence since 2002 and projections for prevalence in England and Wales to 2040: modelling study. bmj. 2017;358.
4. Mitchell SL. Advanced dementia. New England Journal of Medicine. 2015;372(26):2533-40.
5. Banerjee S, Smith S, Lamping D, Harwood R, Foley B, Smith P, et al. Quality of life in dementia: more than just cognition. An analysis of associations with quality of life in dementia. Journal of Neurology, Neurosurgery & Psychiatry. 2006;77(2):146-8.
6. Hall AJ, Febrey S, Goodwin VA. Physical interventions for people with more advanced dementia–a scoping review. BMC geriatrics. 2021;21(1):1-10.

7. Forbes D, Forbes S, Morgan DG, Markle-Reid M, Wood J, Culum I. Physical activity programs for persons with dementia. Cochrane Database of Systematic Reviews. 2008(3).
8. Lamb S, Mistry D, Alleyne S, Atherton N, Brown D, Copsey B, et al. Aerobic and strength training exercise programme for cognitive impairment in people with mild to moderate dementia: the DAPA RCT. Health Technology Assessment. 2018;22(28).
9. Blondell SJ, Hammersley-Mather R, Veerman JL. Does physical activity prevent cognitive decline and dementia?: A systematic review and meta-analysis of longitudinal studies. BMC public health. 2014;14(1):1-12.
10. Groot C, Hooghiemstra AM, Raijmakers PG, van Berckel BN, Scheltens P, Scherder EJ, et al. The effect of physical activity on cognitive function in patients with dementia: a meta-analysis of randomized control trials. Ageing research reviews. 2016;25:13-23.
11. Engel GL. The need for a new medical model: a challenge for biomedicine. Science. 1977;196(4286):129-36.
12. Goodwin VA, Allan LM. 'Mrs Smith has no rehab potential': does rehabilitation have a role in the management of people with dementia? Age and Ageing. 2018;48(1):5-7.
13. Cations M, May N, Crotty M, Low LF, Clemson L, Whitehead C, et al. Health Professional Perspectives on Rehabilitation for People With Dementia. Gerontologist. 2020;60(3):503-12.
14. Hall AJ, Fullam J, Lang IA, Endacott R, Goodwin VA. Community physiotherapy for people with dementia following hip fracture: Fact or fiction? A qualitative study. Dementia. 2020;19(8):2750-60.
15. Scerri A, Innes A, Scerri C. Healthcare professionals' perceived challenges and solutions when providing rehabilitation to persons living with dementia—A scoping review. Journal of Clinical Nursing.n/a(n/a).
16. Mitchell R, Fajardo Pulido D, Ryder T, Norton G, Brodaty H, Draper B, et al. Access to rehabilitation services for older adults living with dementia or in a residential aged care facility following a hip fracture: healthcare professionals' views. Disability and Rehabilitation. 2021;43(6):834-45.
17. McCarroll C, van't Riet C, Halter M. How is the emerging role of domiciliary physiotherapists who treat residents with dementia in nursing homes perceived by allied health professionals? A phenomenological interview study. Health & Social Care in the Community. 2020;28(1):279-90.
18. Borbasi S, Jones J, Lockwood C, Emden C. Health professionals' perspectives of providing care to people with dementia in the acute setting: Toward better practice. Geriatric Nursing. 2006;27(5):300-8.
19. Røsvik J, Rokstad AMM. What are the needs of people with dementia in acute hospital settings, and what interventions are made to meet these needs? A systematic integrative review of the literature. BMC Health Services Research. 2020;20(1):723.
20. Clarke V, Braun V. Thematic analysis. Encyclopedia of critical psychology: Springer; 2014. p. 1947-52.
21. Lynch C. World Alzheimer Report 2019: Attitudes to dementia, a global survey: Public health: Engaging people in ADRD research. Alzheimer's & Dementia. 2020;16:e038255.
22. Zhao W, Moyle W, Wu MLW, Petsky H. Hospital healthcare professionals' knowledge of dementia and attitudes towards dementia care: A cross-sectional study. Journal of Clinical Nursing. 2022;31(13-14):1786-99.
23. Hunter SW, Divine A. Understanding the factors influencing physiotherapists' attitudes towards working with people living with dementia. Physiotherapy Theory and Practice. 2021;37(12):1448-55.
24. Hunter SW, Armstrong J, Silva M, Divine A. Physiotherapy students' attitudes toward working with people with dementia: A cross-sectional survey. Physical & Occupational Therapy In Geriatrics. 2020;38(1):56-66.
25. Cowdell F. Care of older people with dementia in an acute hospital setting. Nursing Standard (through 2013). 2010;24(23):42.

For peer review only

26. Moyle W, Murfield JE, Griffiths SG, Venturato L. Care staff attitudes and experiences of working with older people with dementia. Australasian Journal on Ageing. 2011;30(4):186-90.
27. Houghton C, Murphy K, Brooker D, Casey D. Healthcare staffs' experiences and perceptions of caring for people with dementia in the acute setting: Qualitative evidence synthesis. International Journal of Nursing Studies. 2016;61:104-16.
28. Rokstad AMM, Vatne S, Engedal K, Selbæk G. The role of leadership in the implementation of person-centred care using Dementia Care Mapping: a study in three nursing homes. Journal of Nursing Management. 2015;23(1):15-26.
29. Lynch BM, McCormack B, McCance T. Development of a model of situational leadership in residential care for older people. Journal of Nursing Management. 2011;19(8):1058-69.

For peer review only

Figure title and legend

Figure 1. Theoretical model detailing the interaction of factors and the outcomes for the person with dementia and the HCP

Green denotes positive outcomes, yellow denotes moderate outcomes and red denotes poor outcomes

Values of the HCP	Organisational Culture	Knowledge of the HCP	Outcomes	
			For the person living with dementia	For the HCP
Good	Good	Good	Positive outcomes	Satisfaction and ability to make a positive improvement in care
Good	Good	Poor	Moderate outcomes	Willingness and desire to improve outcomes, but insufficient knowledge to achieve good outcomes
Good	Poor	Poor	Moderate outcomes	Frustration that the HCP values the person with dementia but has neither the positive culture within the organisation or the knowledge to make a positive change.
Good	Poor	Good	Moderate outcomes	Frustration of HCP being unable to achieve good outcomes for the patient as the organisation does not support their interventions
Poor	Poor	Good	Moderate outcomes	Unable to use the knowledge they have in an effective manner
Poor	Poor	Poor	Poor outcomes	Lack of interest or concern from HCP. Skills and knowledge deteriorate further.[i]

Figure 1 - theoretical model detailing the interaction of factors and the outcomes for the person with dementia and the HCP (Green denotes positive outcomes, yellow denotes moderate outcomes and red denotes poor outcomes)

COREQ (COnsolidated criteria for REporting Qualitative research) Checklist

A checklist of items that should be included in reports of qualitative research. You must report the page number in your manuscript where you consider each of the items listed in this checklist. If you have not included this information, either revise your manuscript accordingly before submitting or note N/A.

Topic	Item No.	Guide Questions/Description	Reported on Page No.
**Domain 1: Research team and reflexivity**			
Personal characteristics			
Interviewer/facilitator	1	Which author/s conducted the interview or focus group?	
Credentials	2	What were the researcher's credentials? E.g. PhD, MD	
Occupation	3	What was their occupation at the time of the study?	
Gender	4	Was the researcher male or female?	
Experience and training	5	What experience or training did the researcher have?	
Relationship with participants			
Relationship established	6	Was a relationship established prior to study commencement?	
Participant knowledge of the interviewer	7	What did the participants know about the researcher? e.g. personal goals, reasons for doing the research	
Interviewer characteristics	8	What characteristics were reported about the inter viewer/facilitator? e.g. Bias, assumptions, reasons and interests in the research topic	
**Domain 2: Study design**			
Theoretical framework			
Methodological orientation and Theory	9	What methodological orientation was stated to underpin the study? e.g. grounded theory, discourse analysis, ethnography, phenomenology, content analysis	
Participant selection			
Sampling	10	How were participants selected? e.g. purposive, convenience, consecutive, snowball	
Method of approach	11	How were participants approached? e.g. face-to-face, telephone, mail, email	
Sample size	12	How many participants were in the study?	
Non-participation	13	How many people refused to participate or dropped out? Reasons?	
Setting			
Setting of data collection	14	Where was the data collected? e.g. home, clinic, workplace	
Presence of non-participants	15	Was anyone else present besides the participants and researchers?	
Description of sample	16	What are the important characteristics of the sample? e.g. demographic data, date	
Data collection			
Interview guide	17	Were questions, prompts, guides provided by the authors? Was it pilot tested?	
Repeat interviews	18	Were repeat inter views carried out? If yes, how many?	
Audio/visual recording	19	Did the research use audio or visual recording to collect the data?	
Field notes	20	Were field notes made during and/or after the inter view or focus group?	
Duration	21	What was the duration of the inter views or focus group?	
Data saturation	22	Was data saturation discussed?	
Transcripts returned	23	Were transcripts returned to participants for comment and/or	

For peer review only

Topic	Item No.	Guide Questions/Description	Reported on Page No.
		correction?	
**Domain 3: analysis and findings**			
Data analysis			
Number of data coders	24	How many data coders coded the data?	
Description of the coding tree	25	Did authors provide a description of the coding tree?	
Derivation of themes	26	Were themes identified in advance or derived from the data?	
Software	27	What software, if applicable, was used to manage the data?	
Participant checking	28	Did participants provide feedback on the findings?	
Reporting			
Quotations presented	29	Were participant quotations presented to illustrate the themes/findings? Was each quotation identified? e.g. participant number	
Data and findings consistent	30	Was there consistency between the data presented and the findings?	
Clarity of major themes	31	Were major themes clearly presented in the findings?	
Clarity of minor themes	32	Is there a description of diverse cases or discussion of minor themes?	

Developed from: Tong A, Sainsbury P, Craig J. Consolidated criteria for reporting qualitative research (COREQ): a 32-item checklist for interviews and focus groups. *International Journal for Quality in Health Care*. 2007. Volume 19, Number 6: pp. 349 – 357

Once you have completed this checklist, please save a copy and upload it as part of your submission. DO NOT include this checklist as part of the main manuscript document. It must be uploaded as a separate file.

For peer review only

TOPIC GUIDE

Researcher to introduce self and explain process of focus group, group rules, confidentiality, permission to record, right to withdraw.

Topic	Prompts
**Introduction – each member**<br><br>Please can you tell me a bit about your experience of working with people with advanced dementia and any challenges that you experience supporting them	Profession<br><br>Interest in PwD<br><br>System/organisational pressures<br><br>Individual patient difficulties<br><br>Carer
**Treatments**<br><br>What things are important to ensure that a treatment with a person with advanced dementia is effective?	Who present?<br><br>Involvement of family/carers<br><br>Duration<br><br>Type of intervention<br><br>Patient outcomes / QoL<br><br>Daily routines
**Location**<br><br>What are the key considerations when providing healthcare interventions in a nursing home compared to at home?	Environment<br><br>Formal carers – engagement, education, knowledge<br><br>Equipment
**Communication**<br><br>What factors are important to consider when communicating with a person with advanced dementia?	Body language<br><br>Tone

For peer review only

	Language
**Training/education**<br><br>Therapists with a variety of experience will be treating people with advanced dementia, therefore what education or skills are needed to be able to treat people with advanced dementia?	Undergraduate learning<br><br>Formal learning<br><br>Experiential learning

Is there something else that I have not asked you about, that you would like to tell me about?

Thank you

For peer review only
